# Supplementary material for: A Standardized Clinical Case-Based Assessment for Evaluating Medical Students' Oral Spanish Communication Skills
Source: MedEdPORTAL. 2025 Apr 17;21:11518. doi: 10.15766/mep_2374-8265.11518 (PMC12003672; doi:10.15766/mep_2374-8265.11518)
Supplement: Supplementary file 1 — Precourse Self-Assessment Video.mp4Patient-Provider Interaction Checklist.docxSP Case Spanish.docxSP Case English.docxSP Pilot Case 1 Spanish.docxSP Pilot Case 1 English.docxSP Pilot Case 2 Spanish.docxSP Pilot Case 2 English.docxSP Pilot Case 3 Spanish.docxSP Pilot Case 3 English.docxFacilitators Guide.docx [file mep_2374-8265.11518-s001.zip › B. Patient-Provider Interaction Checklist.docx]

**Appendix B:**

**Patient-Provider Interaction Checklist**

Instructions: Facilitator should use the checklist to score the student communication skills assessment

| Student’s name: | Yes | No | N/A |
| --- | --- | --- | --- |
| 1. Greet the patient appropriately for the time of day (e.g. good afternoon, good morning, good evening) |  |  |  |
| 2. Introduces themselves and explains their role (e.g. I'm John Smith, medical student) |  |  |  |
| 3. Ask for the patient's first and last name; ask the patient how they prefer to be called (e.g. Mr. Carlos, Mrs. Perez, Ms. Rosa) |  |  |  |
| 4. Check the patient's identity (e.g. date of birth, age) |  |  |  |
| 5. Start with small talk (e.g. Did you have any trouble finding parking?) |  |  |  |
| 6. Ask open-ended questions (e.g. How are you?) |  |  |  |
| 7. Question about the chief concern (e.g. How can I help you? Why did you come to see us?) |  |  |  |
| 8. Ask for details about the current illness (e.g. Tell me more about the chest pain? When did this chest pain start?) |  |  |  |
| 9. Ask what the patient attributes their health problems to (e.g. Why do you think they have this chest pain? What do you blame for this chest pain?) Applies to any health problems that arise in the interview |  |  |  |
| 10. Ask about the impact that the current disease has on the life of the  patient (e.g. Does the pain affect your sleep, work, or family?). Applies to any problems that arise in the interview (e.g. Is alcohol consumption affecting you at work?) |  |  |  |
| 11. Reviews steps of the visit with the patient  (e.g. I'm going to ask you questions first, then I'm going to examine you. And at the end we will talk about what we're going to do with your problem, what do you think?) |  |  |  |
| 12. Ask for details about medical and surgical history  (e.g. When were you diagnosed with diabetes? Have you ever had surgery?) |  |  |  |
| 13. Ask about Medications, Allergies, and Adverse Reactions (e.g. Do you take any medicines? Do you have an allergy to any medicines?) |  |  |  |
| 14. Ask for alternative or complementary medicines (e.g. Do you take any vitamins? Do you take medicinal herbs? Do you take any home remedy?) |  |  |  |
| 15. Ask for details about family history  (e.g. Has anyone in your family had any health problems, such as cancer or diabetes?) |  |  |  |
| 16. Ask for signs and symptoms (systems review) of at least  two systems (e.g. Have you had palpitations? Have you had shortness of breath? Have you had vision problems? Why do you wear glasses?) |  |  |  |
| 17. Ask about habits and behaviors related to health (e.g. Do you smoke cigarettes, pipes, or cigars? Do you drink alcohol? How many glasses of wine do you drink? Do you do physical activity?) |  |  |  |
| 18. Ask details about the social background (e.g. What do you do for work? Who do you live with? How many children do you have?) |  |  |  |
| 19. Try to establish a good relationship with the patient (rapport) and show empathy (e.g. respond to verbal/nonverbal expression of feelings and emotions, either by making an empathetic comment, “I'm so sorry”, or a thumbs-up gesture for something positive, smiling, etc.) |  |  |  |
| 20. Close the interview in a respectful and calm way (e.g. nice to meet you, good afternoon, see you later, goodbye) |  |  |  |
|  |  |  |  |
| **Question to ask the student during feedback** |  |  |  |
| 1. What did you learn from this patient? |  |  |  |
| 2. Any belief or concept that caught your attention? |  |  |  |
