# Supplementary material for: A Standardized Clinical Case-Based Assessment for Evaluating Medical Students' Oral Spanish Communication Skills
Source: MedEdPORTAL. 2025 Apr 17;21:11518. doi: 10.15766/mep_2374-8265.11518 (PMC12003672; doi:10.15766/mep_2374-8265.11518)
Supplement: Supplementary file 1 — Precourse Self-Assessment Video.mp4Patient-Provider Interaction Checklist.docxSP Case Spanish.docxSP Case English.docxSP Pilot Case 1 Spanish.docxSP Pilot Case 1 English.docxSP Pilot Case 2 Spanish.docxSP Pilot Case 2 English.docxSP Pilot Case 3 Spanish.docxSP Pilot Case 3 English.docxFacilitators Guide.docx [file mep_2374-8265.11518-s001.zip › C. SP Case Spanish.docx]

Appendix C: Standardized Patient Case Development Tool Spanish

Instructions: Facilitator and Standardized Patient should use the Standardized Patient script to conduct the student communication skills assessment

Primary Case Author: Silvana Bonilla, MD

Secondary Case Author: Alejandro Diaz, MD

Name of Case: Dolor en el pecho

Name of Educational and/or Assessment Activity: Evaluación de fin de curso de Español Médico

Type and Level of Learner: Estudiante de Español Médico de nivel Intermedio a Avanzado

Patient Name: Camilo/a Pérez

Chief Concern: Dolor en el pecho

Most Likely Diagnosis and Differential with Rationale from History and/or Physical Exam: El diagnóstico más probable en este paciente es costocondritis, sugerido por el reciente aumento en su actividad física debido a un nuevo supervisor en el trabajo, lo que ha generado una carga laboral más pesada. Siendo trabajador de construcción (o empleada doméstica), el esfuerzo físico podría fácilmente contribuir a la inflamación del cartílago costal. El dolor del paciente es reproducible mediante la palpación y se alivia con medicamentos para el dolor, lo que respalda aún más este diagnóstico.El diagnóstico diferencial incluye problemas médicos como un ataque al corazón. Sin embargo, el paciente no presenta signos clave como irradiación del dolor, diaforesis, náuseas o mareos. El dolor se agrava con el esfuerzo, pero no se alivia con el descanso, lo que es típico en la angina o el infarto de miocardio. Otra consideración es un ataque de asma, dado el historial de asma del paciente. No obstante, la ausencia de síntomas respiratorios como sibilancias, dificultad para respirar o tos hace que esta posibilidad sea menos probable. La enfermedad por reflujo gastroesofágico (ERGE) también podría ser un diagnóstico potencial, pero los síntomas del paciente no se desencadenan por la ingesta de alimentos, ni hay otros síntomas gastrointestinales relevantes como acidez o regurgitación. Además, el paciente no presenta factores de riesgo ni síntomas constitucionales como aumento de peso repentino o cambios dietéticos que sugieran que la ERGE sea la causa principal. Por lo tanto, la presentación clínica, incluyendo los síntomas negativos pertinentes, confirman que la costocondritis sea el diagnóstico más probable.

Domains: Check all that apply

- Professionalism
- Communication and Interpersonal Skills
- Medical History
- Physical Exam
- Shared Decision-Making
- Patient Education
- Clinical Reasoning
- Documentation
- Handoff
- Presentation
- Other:

Case Objectives: Please list specific objectives for each of the domains you have checked above

1. Profesionalismo: Demuestra respeto por las preocupaciones del paciente manteniendo un enfoque empático y libre de juicios durante toda la evaluación del dolor torácico.
2. Habilidades de comunicación e interpersonales: Establece una buena relación escuchando activamente la descripción de los síntomas del paciente y asegurándose de que se sienta escuchado y comprendido.
3. Historia médica: Obtiene una historia clínica completa enfocándose en el inicio, la duración y la naturaleza del dolor torácico, especialmente en relación con la actividad física, la sensibilidad al tacto y la respuesta a medicamentos para el dolor. Explora antecedentes médicos relevantes, como asma, trastornos gastrointestinales o episodios previos de dolor torácico, para ayudar en el diagnóstico diferencial. Obtiene síntomas negativos pertinentes, como dolor torácico con esfuerzo fisico, dificultad respiratoria o desencadenantes gastrointestinales, para descartar otras problemas médicos graves.
4. Razonamiento clínico: Demuestra razonamiento clínico priorizando la costocondritis como el diagnóstico más probable, basándose en el reciente aumento de la actividad física del paciente y la reproducibilidad del dolor mediante palpación. Resalta la importancia de seguir en contacto con el proveedor de atención primaria para monitorear la resolución de los síntomas.

Standardized Patient Script:

| SETTING: outpatient, in patient, ED, home, nursing home, rehab, group, etc. | Clínica de atención de urgencias a las 2 PM. |
| --- | --- |
| PATIENT PROFILE: Information about the “patient” that helps select an SP and helps the learner get an understanding of them as a person. SP will know more information about the patient than learner will ever ask but allows SP to portray a fully developed patient personality. If none of the items below are particulars for the case, please write “Any answer acceptable.” | |
| Age range | 54 años. |
| Religious/spiritual background | Usted es católico, y va a la iglesia dos o tres veces al año, como por ejemplo para semana santa y Navidad. |
| Sex (e.g. male, female, intersex, transwoman, transman) | Cualquier respuesta es aceptable. |
| Sexual orientation (e.g. heterosexual, lesbian, gay, bisexual, pansexual, queer, asexual) | Cualquier respuesta es aceptable. |
| Gender expression (e.g. man, woman, genderqueer) | Cualquier respuesta es aceptable. |
| Race and ethnicity (e.g. to promote educational diversity, we use a diverse pool of SPs.) | Persona hispana/latina. |
| Physical description (e.g. BMI, height range) | Cualquier respuesta es aceptable. |
| Physical limitations | Cualquier respuesta es aceptable. |
| Patient appearance (e.g. disheveled, hospital gown, business casual, casual) | Estás alerta y vestido con ropa informal para trabajar. |
| Moulage + location (e.g. none, bruises, scars, body piercing, tattoos) | Cualquier respuesta es aceptable. |
| Affect (e.g. pleasant, cooperative) | Estás visiblemente incómodo por el dolor, pero cooperativo. |
| Family group (e.g. who is family, who they live with) | Vive con su cónyuge y una hija de 22 años que está terminando la universidad. |
| Education | Terminó la escuela secundaria. |
| Level of health literacy | Nivel de alfabetización en salud bajo a intermedio. |
| Employment, if any - present and past, noting any current stresses | Trabaja en una compañía de construcción (voluntario varón) o de limpieza de casas (voluntaria mujer). Tiene un nuevo supervisor en su trabajo y se siente más agobiado con él, ya que ha estado más encima de usted, objetando su manera de trabajar. |
| Home/homeless - type of dwelling, number of stories, owned or rented | Inmigró de su país natal Venezuela hace 15 años. Vive en Chelsea, MA. |
| Financial situation - any current stresses | Cualquier respuesta es aceptable. |
| Insurance status (e.g. un/under/insured, public/private, HMO/PPO) | Cualquier respuesta es aceptable. |
| Habits (i.e., diet, exercise, caffeine, smoking, alcohol, drugs) | No fuma, toma 2 botellas de cerveza los fines de semana. Nunca ha tenido problemas por el alcohol en la casa o el trabajo. Niega usar otras drogas. Camina dos veces semana con su esposa por recomendación de su médico de cabecera. Come carnes rojas 3 veces por semana y no mucha verdura. En el trabajo comen sándwiches o pizza, pero también come con poca sal por recomendación de su médico de cabecera. |
| Activities (i.e., hobbies, sports, clubs, friends) | Disfruta pasar tiempo con su familia extendida que viven cerca. |
| Typical day - what is the usual daily routine | Te levantas temprano y bebes una taza de té de manzanilla todas las mañanas antes de ir a trabajar. Trabajas entre las 8:00 am y las 5:00 pm la mayoría de los días. Después del trabajo, preparas la comida con su cónyuge y miran televisión juntos antes de ir a dormir. |

| CASE INFORMATION | |
| --- | --- |
| Chief Concern: What the patient will say when greeted by the student. The patient’s primary reason for seeking medical care often stated in their own words. | “Me duele el pecho.” |
| Additional Concerns: Other, if any, concerns the patient has today (i.e., symptoms, requests, expectations, etc.) that will become part of set agenda. | Ninguna. |
| THE PATIENT’S STORY: The SP will be asked to tell their symptom story and the personal and emotional impact for each of their concerns. You will want to write this in the patient’s voice. The symptom story should be able to answer this question: “Tell me more about [chief concern/additional concern], starting at the beginning and bringing me up to now.”  The personal context should be able to answer questions concerning the broader personal/psychosocial context of symptoms, especially the patient’s beliefs/attributions.  The emotional context should be able to ask how are you doing with this, how does this make you feel, how has this affected you emotionally? IMPACT: How has this affected your life? How has this been for your family? | “Tengo dolor aquí (el paciente señala la mitad del pecho, al lado derecho del esternón). El dolor comenzó hace 4 días después que salí a la calle sin chaqueta; era un día muy frío y creo que “tomé frío”. El dolor se siente como una presión constante y ha empeorado. Se extiende hacia la derecha y duele especialmente si muevo el brazo derecho o lo presiono, así que trato de mantener el hombro quieto. Tomo Tylenol que me ayuda un poco, pero nunca antes había tenido este tipo de dolor y tengo miedo. Si el dolor continúa así, no sé si podré seguir yendo a mi trabajo para mantener a mi familia.” |
| HISTORY OF PRESENT ILLNESS: Although some of the HPI will be given in the patient’s symptom story, the learners will expand the story during the direct question section. Below, describe the detailed history, usually about the chief concern, which the student must develop in order to make a useful assessment of the problem: | |
| Onset (when; gradual or sudden) | Hace 4 días; inicio rápido. |
| Setting (what was going on or where was patient when symptoms first noticed?) | El dolor empezó después de salir sin chaqueta en un día muy frío. |
| Duration (how long) | 4 días hasta ahora. |
| Time relationships (frequency, constant or intermittent) | Constante. |
| Location | El dolor se localiza en el medio del pecho, en el lado derecho del hueso medio del pecho (esternón). |
| Radiation | Irradiando hacia el lado derecho. |
| Quality | Presión. |
| Amount | Intensidad inicial de 3/10 pero en el último día ha aumentado hasta 6/10. |
| Aggravated by what | Si presionas la zona dolorida, notarás que el dolor aumenta. El dolor también empeora cuando mueves el brazo derecho. |
| Relieved by what | El dolor mejora parcialmente con 2 comprimidos de 500 mg de paracetamol (Tylenol). El dolor es menor cuando no se mueve el hombro. |
| Associated with what | Ninguna. |
| Attitude (what does the patient think is the problem, and how do they feel about it) | No has sentido este tipo de dolor en el pasado. Crees que tienes este dolor porque "tomé frío" y expresas el dolor poniéndote la mano en el pecho. |
| Overall course | El dolor está empeorando. |
| REVIEW OF SYSTEMS: Significant positives and negatives | |
| NEGATIVES | POSITIVES |
| Niega náuseas o vómitos, agruras, eructos, tos, pérdida de la fuerza o sensibilidad. No ha tenido dificultad para respirar. | Dolor en el pecho. |
| Niega dolor de garganta, dolor abdominal, diarrea, pérdida de peso, sudoración, fiebre, palpitaciones, arritmias o problemas del corazón. | Dolor de mandíbula desde hace 2 meses (bruxismo). |
| No ha tenido problemas de los riñones o para orinar. |  |
| Niega cambios de visión, cambios de audición, o dolor en el cuello. No ha sufrido caídas ni convulsiones. Niega cambios en la piel. |  |
|  |  |
| Past medical history |  |
| Medication allergies (name and reaction) | Ninguna. |
| Environmental allergies (name and reaction) | Ninguna. |
| Illnesses | Presión arterial alta – Usted tiene presión arterial alta desde hace 4 años. La última vez que se chequeó los valores eran 13 sobre 8 (esto es 130/80). También come con poca sal. No tiene antecedentes de colesterol alto o problemas del corazón. No se ha evaluado la vista o los riñones. Su doctor de cabecera le pidió una ecografía del corazón que salió bien.  Asma – Asma leve, empezó desde los 10 años, pero de adulto le afecta poco. Usa un inhalador para abrir los bronquios ocasionalmente. Nunca estuvo hospitalizado por asma. Una vez, a los 13 años, fue a la emergencia. La presión arterial y el asma los maneja su médico de cabecera. |
| Vaccinations | Al día con todas las vacunas. |
| Surgeries | Apendectomía. |
| Accidents/injuries/trauma | Ninguna. |
| Hospitalization | 22 años por apendicitis. Operado en Venezuela. No tuvo complicaciones. |
|  | |
| Inclusive sexual and reproductive history | |
| Sexual practices  Sexual partners  Protection: Use of safer sex practices  Use of birth control if appropriate  Risk of intimate partner violence | Cualquier respuesta es aceptable. |
| OB/GYN history | Cualquier respuesta es aceptable. |
| Medications | Atenolol una pastilla de 50 mg al día para controlar la presión, buena adherencia al tratamiento. Un inhalador para abrir los bronquios ocasionalmente. No usa vitaminas ni remedios caseros. Toma te de manzanilla todas las mañanas. |
| Immunizations | - Tétano - Gripe - Hepatitis - Vacuna antineumocócica - VPH - Otros: COVID |
| Tobacco products   - Cigarrillos - Puros - Pipas - Masticables - Cigarrillos electrónicos | - Nunca - Pasado – año de inicio/año de abandono - Actual   - Cantidad   - # de años |
| Alcohol   - Cerveza - Vino - Licor - Otros | - Nunca - Pasado – año de inicio/año de abandono - Actual   - 2 botellas los fines de semana   - 35 años |
| Drugs   - Marihuana - Cocaína - Heroína - Metanfetamina - Drogas intravenosas - Inhalantes - Otros | - Nunca - Pasado – año de inicio/año de abandono - Actual   - Cantidad   - # de años |
| Diet (describe) | Come carnes rojas 3 veces por semana y no mucha verdura. En el trabajo comen sándwiches o pizza, pero también come con poca sal por recomendación de su médico de cabecera. |
| Exercise (describe) | Camina dos veces semana con su esposa por recomendación de su médico de cabecera. |
| List any other important social history or information important to this case | Ninguna. |
| Family history |  |
| Mother, father, siblings, grandparents, and other significant findings | Sus padres están vivos. Su padre (85 años) tuvo un infarto de miocardio (ataque al corazón) a los 55 años. Su madre (80 años) tuvo cáncer de cérvix (cuello del útero), a los 45 años. Se lo encontraron a tiempo y le hicieron una operación. También ella tiene asma leve desde la infancia, usa un inhalador. Usted tiene una hermana, Sara, de 52 años que también tiene la presión arterial alta. Usted no recuerda nada de sus abuelos paternos ni maternos. |
|  |  |
| Physical Exam - List exam maneuvers expected for this case and any abnormal findings that SP will simulate. (tenderness, hyper-hypo reflex, rebound, weakness, etc.)  Camilo/a se sentará inclinado hacia adelante y colocará su mano sobre su pecho para expresar dolor durante el encuentro. También parecerá fatigado durante todo el encuentro.  No se realizará ningún examen físico durante este caso. | |
| PHYSICAL EXAM FINDINGS |  |
| 1. Written in layperson’s terms |  |
| 1. General appearance - affect, appearance, position of patient at opening (i.e., sitting, lying down, holding abdomen, etc.) | Cuando el estudiante se una a la videollamada deberá estar sentado en una silla vistiendo su ropa habitual. |
| 1. Vital signs | Temperatura: 99° F  Pulso: 75 bpm  Presión arterial: 132/64  Frecuencia respiratoria: 18 |
| 1. Specific findings and affect | Camilo/a parecerá fatigado durante todo el encuentro. |
| 1. Response to certain physical movements | Camilo/a colocará su mano sobre su pecho para expresar dolor durante el encuentro. |
|  |  |
| DIAGNOSIS AND DIFFERENTIAL |  |
| Diagnosis with support from positive and negative history and PE findings | Costocondritis |
| Differential with support from positive and negative history and PE findings | Ataque al corazón, Ataque de asma, Enfermedad por reflujo gastroesofágico (ERGE) |
|  |  |
| MANAGEMENT OR DIAGNOSTIC PLAN | Tranquiliza al paciente respecto al diagnóstico de costocondritis. Recomienda un AINE, como ibuprofeno, 200-600 mg dos veces al día para un mejor control del dolor. Se asegura de que el paciente tenga una cita de seguimiento con su proveedor de atención primaria dentro de una semana para monitorear la resolución de los síntomas. |
|  |  |
| PROFESSIONALISM ISSUES OR CHALLENGES | Competencia cultural. |
