# Supplementary material for: A Standardized Clinical Case-Based Assessment for Evaluating Medical Students' Oral Spanish Communication Skills
Source: MedEdPORTAL. 2025 Apr 17;21:11518. doi: 10.15766/mep_2374-8265.11518 (PMC12003672; doi:10.15766/mep_2374-8265.11518)
Supplement: Supplementary file 1 — Precourse Self-Assessment Video.mp4Patient-Provider Interaction Checklist.docxSP Case Spanish.docxSP Case English.docxSP Pilot Case 1 Spanish.docxSP Pilot Case 1 English.docxSP Pilot Case 2 Spanish.docxSP Pilot Case 2 English.docxSP Pilot Case 3 Spanish.docxSP Pilot Case 3 English.docxFacilitators Guide.docx [file mep_2374-8265.11518-s001.zip › D. SP Case English.docx]

Appendix D: Standardized Patient Case Development Tool English

Instructions: Facilitator and Standardized Patient should use the Standardized Patient script to conduct the student communication skills assessment

Primary Case Author: Silvana Bonilla, MD

Secondary Case Author: Alejandro Diaz, MD

Name of Case: Chest pain

Name of Educational and/or Assessment Activity: End-of-Course Evaluation for Medical Spanish Course

Type and Level of Learner: Intermediate to Advanced Medical Spanish Student

Patient Name: Camilo/a Pérez

Chief Concern: Chest pain

Most Likely Diagnosis and Differential with Rationale from History and/or Physical Exam: The most likely diagnosis for this patient is costochondritis, which is suggested by the patient's recent increase in physical activity due to a new supervisor at work, leading to a heavier workload. As a construction worker (or house cleaner), the physical strain could easily contribute to inflammation of the costal cartilage. The patient's pain is reproducible by palpation and alleviated by over-the-counter pain medications, further supporting this diagnosis. The differential diagnosis includes conditions such as a heart attack. However, the patient lacks key signs such as radiation of pain, diaphoresis, nausea, and dizziness. The pain is exacerbated by exertion but not relieved by rest, which is typical in angina or myocardial infarction. Another consideration is an asthma attack, given the patient’s history of asthma. However, the absence of respiratory symptoms like wheezing, shortness of breath, or cough makes this less likely. Gastroesophageal reflux disease (GERD) could also be a potential diagnosis, but the patient's symptoms are not triggered by food intake, and there are no other relevant gastrointestinal symptoms such as heartburn or regurgitation. Additionally, the patient does not exhibit risk factors or constitutional symptoms like sudden weight gain or dietary changes that might suggest GERD as a primary cause. Therefore, the clinical presentation, including pertinent negatives, strongly supports costochondritis as the most likely diagnosis.

Domains: Check all that apply

- Professionalism
- Communication and Interpersonal Skills
- Medical History
- Physical Exam
- Shared Decision-Making
- Patient Education
- Clinical Reasoning
- Documentation
- Handoff
- Presentation
- Other:

Case Objectives: Please list specific objectives for each of the domains you have checked above

1. Professionalism: Demonstrate respect for the patient’s concerns by maintaining a non-judgmental and empathetic approach throughout the evaluation of chest pain.
2. Communication and Interpersonal Skills: Establish rapport by actively listening to the patient's description of symptoms and ensuring they feel heard and understood.
3. Medical History: Elicit a comprehensive history focusing on the onset, duration, and character of the chest pain, particularly about physical activity, touch sensitivity, and response to over-the-counter medications. Explore relevant past medical history, including conditions such as asthma, gastrointestinal disorders, or any previous episodes of chest pain, to aid in differential diagnosis. Obtain pertinent negatives, such as exertional chest pain, respiratory distress, or gastrointestinal triggers, to rule out other serious conditions.
4. Clinical Reasoning: Demonstrate clinical reasoning by prioritizing costochondritis as the most likely diagnosis based on the patient’s recent increase in physical activity and reproducibility of pain on palpation. Stress the importance of following up with the primary care provider to monitor for resolution of symptoms.

Standardized Patient Script:

| SETTING: outpatient, in patient, ED, home, nursing home, rehab, group, etc. | Urgent care at 2PM. |
| --- | --- |
| PATIENT PROFILE: Information about the “patient” that helps select an SP and helps the learner get an understanding of them as a person. SP will know more information about the patient than learner will ever ask but allows SP to portray a fully developed patient personality. If none of the items below are particulars for the case, please write “Any answer acceptable.” | |
| Age range | 54 years old. |
| Religious/spiritual background | You are Catholic and you go to church two or three times per year for holidays, such as Easter and Christmas. |
| Sex (e.g. male, female, intersex, transwoman, transman) | Any answer acceptable. |
| Sexual orientation (e.g. heterosexual, lesbian, gay, bisexual, pansexual, queer, asexual) | Any answer acceptable. |
| Gender expression (e.g. man, woman, genderqueer) | Any answer acceptable. |
| Race and ethnicity (e.g. to promote educational diversity, we use a diverse pool of SPs.) | Hispanic/Latinx. |
| Physical description (e.g. BMI, height range) | Any answer acceptable. |
| Physical limitations | Any answer acceptable. |
| Patient appearance (e.g. disheveled, hospital gown, business casual, casual) | You are alert and dressed in casual clothing for work. |
| Moulage + location (e.g. none, bruises, scars, body piercing, tattoos) | Any answer acceptable. |
| Affect (e.g. pleasant, cooperative) | You are visibly uncomfortable from the pain but cooperative. |
| Family group (e.g. who is family, who they live with) | You live with your spouse and a 22-year-old daughter who is finishing college. |
| Education | Completed high school. |
| Level of health literacy | Low to intermediate health literacy. |
| Employment, if any - present and past, noting any current stresses | You work for a construction company or as a house cleaner (SP volunteer preference). You have a new supervisor at your job and you feel more burdened with him, as he has been criticizing the way you work. |
| Home/homeless - type of dwelling, number of stories, owned or rented | You emigrated from Venezuela 15 years ago. You now live in Chelsea, MA in a second story apartment you rent. |
| Financial situation - any current stresses | Any answer acceptable. |
| Insurance status (e.g. un/under/insured, public/private, HMO/PPO) | Any answer acceptable. |
| Habits (i.e., diet, exercise, caffeine, smoking, alcohol, drugs) | You do not smoke; you drink 2 bottles of beer on weekends. You have never had problems with alcohol at home or work. You do not use any other drugs. You walk twice a week with your spouse on the recommendation of your primary care doctor. You eat red meat 3 times a week and not many vegetables. At work, you eat sandwiches or pizza but otherwise you eat your meals with very little salt as instructed by your primary care doctor. |
| Activities (i.e., hobbies, sports, clubs, friends) | You enjoy spending time with your extended family who live in the area. |
| Typical day - what is the usual daily routine | You rise early and drink a cup of chamomile tea each morning before going to work. You work between 8am and 5pm most days. After work, you prepare food with your spouse and watch TV together before going to bed. |

| CASE INFORMATION | |
| --- | --- |
| Chief Concern: What the patient will say when greeted by the student. The patient’s primary reason for seeking medical care often stated in their own words. | “My chest hurts.” |
| Additional Concerns: Other, if any, concerns the patient has today (i.e., symptoms, requests, expectations, etc.) that will become part of set agenda. | None. |
| THE PATIENT’S STORY: The SP will be asked to tell their symptom story and the personal and emotional impact for each of their concerns. You will want to write this in the patient’s voice. The symptom story should be able to answer this question: “Tell me more about [chief concern/additional concern], starting at the beginning and bringing me up to now.”  The personal context should be able to answer questions concerning the broader personal/psychosocial context of symptoms, especially the patient’s beliefs/attributions.  The emotional context should be able to ask how are you doing with this, how does this make you feel, how has this affected you emotionally? IMPACT: How has this affected your life? How has this been for your family? | “I have been having pain here (patient points to the middle of the chest, on the right side of the sternum). This started 4 days ago after I went outside without a jacket; it was a very cold day and I think I caught a cold. The pain feels like a constant pressure and has become worse. It spreads towards the right and especially hurts if I move my right arm or press on it, so I try to keep my shoulder still. I have been taking Tylenol which helps some, but I’ve never had this type of pain in the past and am scared. If the pain continues like this, I don’t know if I can keep going to my job to support my family.” |
| HISTORY OF PRESENT ILLNESS: Although some of the HPI will be given in the patient’s symptom story, the learners will expand the story during the direct question section. Below, describe the detailed history, usually about the chief concern, which the student must develop in order to make a useful assessment of the problem: | |
| Onset (when; gradual or sudden) | 4 days ago; rapid onset. |
| Setting (what was going on or where was patient when symptoms first noticed?) | The pain started after you went outside without a jacket on a very cold day. |
| Duration (how long) | 4 days so far. |
| Time relationships (frequency, constant or intermittent) | Constant. |
| Location | The pain is located in the middle of the chest, on the right side of the middle chest bone (sternum). |
| Radiation | Radiating to the right side. |
| Quality | Pressure. |
| Amount | Initial intensity of 3/10 but in the last day has increased to 6/10. |
| Aggravated by what | If you press on the area of pain, you notice that the pain increases. The pain also gets worse when you move with your right arm. |
| Relieved by what | Pain partially improves with 2 tablets of 500mg of acetaminophen (Tylenol). The pain is less when the shoulder is not moved. |
| Associated with what | None. |
| Attitude (what does the patient think is the problem, and how do they feel about it) | You have not experienced this type of pain in the past. You think you have this pain because you "caught a cold" and express the pain by putting your hand on your chest. |
| Overall course | Pain is worsening. |
| REVIEW OF SYSTEMS: Significant positives and negatives | |
| NEGATIVES | POSITIVES |
| Denies nausea or vomiting, cough, fatigue, or shortness of breath. | Chest pain. |
| Denies muscle pain, sore throat, abdominal pain, diarrhea, weight loss, sweating, fever, palpitations, arrhythmias, or heart problems. | Jaw pain for 2 months (bruxism). |
| Denies kidney or urination problems. |  |
| Denies vision changes, hearing changes, neck pain, falls or convulsions. Denies skin changes. |  |
|  |  |
| Past medical history |  |
| Medication allergies (name and reaction) | None. |
| Environmental allergies (name and reaction) | None. |
| Illnesses | Hypertension – You have had high blood pressure for 4 years. The last time the values were 130/80. You have no history of high cholesterol or heart problems. Vision or kidneys have not been evaluated. Your primary care doctor asked for an ultrasound of the heart which to your knowledge was normal.  Asthma – You have been diagnosed with mild asthma since age 10 years. The symptoms were moderate during childhood but improved as you grew older. You use an inhaler occasionally. You have never been hospitalized for asthma. Once, at age 13, you went to the emergency room but were not hospitalized. Blood pressure and asthma are managed by your primary care doctor. |
| Vaccinations | Up to date with all vaccines. |
| Surgeries | Appendectomy. |
| Accidents/injuries/trauma | None. |
| Hospitalization | Appendicitis at 22 years old in Venezuela. No complications. |
|  | |
| Inclusive sexual and reproductive history | |
| Sexual practices  Sexual partners  Protection: Use of safer sex practices  Use of birth control if appropriate  Risk of intimate partner violence | Any answer acceptable. |
| OB/GYN history | Any answer acceptable. |
| Medications | Atenolol 1 tablet 50 mg daily to control your blood pressure. You use an inhaler occasionally for your asthma. You do not take vitamins or home remedies. You drink chamomile tea every morning. |
| Immunizations | - Tetanus - Flu - Hepatitis - Pneumovax - HPV - Other: COVID |
| Tobacco products   - Cigarettes - Cigar - Pipe - Chew - E-cigarettes | - Never - Past - year started/year quit - Current   - Quantity   - # of years |
| Alcohol   - Beer - Wine - Liquor - Other | - Never - Past - year started/year quit - Current   - 2 bottles on weekends   - 35 years |
| Drugs   - Weed - Cocaine - Heroin - Meth - IV - Inhalants - Other | - Never - Past - year started/year quit - Current   - Quantity   - # of years |
| Diet (describe) | You eat red meat 3 times a week and not many vegetables. At work, you eat sandwiches or pizza but otherwise you eat your meals with very little salt as instructed by your primary care doctor. |
| Exercise (describe) | You walk twice a week with your spouse on the recommendation of your primary care doctor. |
| List any other important social history or information important to this case | None. |
| Family history |  |
| Mother, father, siblings, grandparents, and other significant findings | Your parents are alive. Your father (85 years old) had a heart attack at age 55. Your mother (80 years old) had cervical cancer at age 45. She was diagnosed early and she was able to have a surgery to remove the cancer. She also has mild asthma since childhood and she uses an inhaler. You have a sister, Sara (52 years old), who also has high blood pressure. You don't remember anything about your paternal or maternal grandparents. |
|  |  |
| Physical Exam - List exam maneuvers expected for this case and any abnormal findings that SP will simulate. (tenderness, hyper-hypo reflex, rebound, weakness, etc.)  Camilo/a will sit leaning forward and will place their hand over their chest to express pain throughout the encounter. They will also appear fatigued throughout the encounter.  There is no physical examination during this case. | |
| PHYSICAL EXAM FINDINGS |  |
| 1. Written in layperson’s terms |  |
| 1. General appearance - affect, appearance, position of patient at opening (i.e., sitting, lying down, holding abdomen, etc.) | When the student joins the video call you should be sitting in a chair wearing your regular clothes. |
| 1. Vital signs | T: 99° F  Pulse: 75 bpm  BP: 132/64  RR: 18 |
| 1. Specific findings and affect | Camilo/a will appear fatigued throughout the encounter. |
| 1. Response to certain physical movements | Camilo/a will place their hand over their chest to express pain throughout the encounter. |
|  |  |
| DIAGNOSIS AND DIFFERENTIAL |  |
| Diagnosis with support from positive and negative history and PE findings | Costochondritis |
| Differential with support from positive and negative history and PE findings | Heart Attack, Asthma exacerbation, GERD |
|  |  |
| MANAGEMENT OR DIAGNOSTIC PLAN | Reassure the patient of the diagnosis of costochondritis. Recommend an NSAID, such as ibuprofen, 200-600 mg twice a day for better control of pain. Ensure follow-up with the primary care provider within a week to monitor for resolution of symptoms. |
|  |  |
| PROFESSIONALISM ISSUES OR CHALLENGES | Cultural competency. |
