# Supplementary material for: A Standardized Clinical Case-Based Assessment for Evaluating Medical Students' Oral Spanish Communication Skills
Source: MedEdPORTAL. 2025 Apr 17;21:11518. doi: 10.15766/mep_2374-8265.11518 (PMC12003672; doi:10.15766/mep_2374-8265.11518)
Supplement: Supplementary file 1 — Precourse Self-Assessment Video.mp4Patient-Provider Interaction Checklist.docxSP Case Spanish.docxSP Case English.docxSP Pilot Case 1 Spanish.docxSP Pilot Case 1 English.docxSP Pilot Case 2 Spanish.docxSP Pilot Case 2 English.docxSP Pilot Case 3 Spanish.docxSP Pilot Case 3 English.docxFacilitators Guide.docx [file mep_2374-8265.11518-s001.zip › E. SP Pilot Case 1 Spanish.docx]

Appendix E: Standardized Patient Development Tool Pilot Case 1 Spanish

Instructions: Facilitator and Standardized Patient should use the Standardized Patient script to conduct the student communication skills assessment

Primary Case Author: Cristina Aguayo-Mazzucato, MD PhD

Secondary Case Author: Brandon Martel

Name of Case: Problemas menstruales

Name of Educational and/or Assessment Activity: Caso piloto de español medico

Type and Level of Learner: Estudiante de español médico de nivel intermedio a avanzado

Patient Name: María Cortinez

Chief Concern: Ausencia de períodos menstruales

Most Likely Diagnosis and Differential with Rationale from History and/or Physical Exam: El diagnóstico más probable es Síndrome de Ovario Poliquístico (SOP). La paciente presenta amenorrea secundaria, aumento de peso, hirsutismo (vellos en la barbilla) y posiblemente galactorrea (secreción de leche). Estos síntomas son compatibles con el SOP, un trastorno endocrino común que puede causar ciclos menstruales irregulares y un aumento de hormonas androgénicas. Otras problemas médicos en el diagnóstico diferencial son: Hiperprolactinemia, posiblemente causada por un adenoma hipofisario (prolactinoma) u otras causas hormonales. Hipotiroidismo, que puede causar amenorrea, galactorrea y aumento de peso. Una disminución en la función tiroidea afecta los ciclos menstruales y el metabolismo, lo que explica algunos de los síntomas de la paciente. Insuficiencia ovárica prematura (menopausia precoz), que podría estar relacionada con factores genéticos o autoinmunes. Causas secundarias de amenorrea (estrés, ejercicio excesivo). Aunque la paciente lleva un estilo de vida saludable, el estrés laboral y la actividad física (correr varias veces a la semana) podrían contribuir a la alteración de los ciclos menstruales. Sin embargo, otros síntomas como la galactorrea sugieren una causa más hormonal.

Domains: Check all that apply

- Professionalism
- Communication and Interpersonal Skills
- Medical History
- Physical Exam
- Shared Decision-Making
- Patient Education
- Clinical Reasoning
- Documentation
- Handoff
- Presentation
- Other:

Case Objectives: Please list specific objectives for each of the domains you have checked above

1. Demostrar competencia cultural siendo sensible al contexto y las elecciones de estilo de vida de la paciente, como su dieta vegetariana y sus hábitos de ejercicio, sin hacer suposiciones.
2. Establecer una buen rapport con la paciente escuchándola activamente, abordando sus preocupaciones sobre la menopausia y los desequilibrios hormonales, y creando un ambiente cómodo para una comunicación abierta.
3. Tomar una historia clínica completa enfocada en el ciclo menstrual, los cambios hormonales y los factores de estilo de vida de la paciente, prestando especial atención a cualquier antecedente familiar de problemas endocrinos o reproductivos.

Standardized Patient Script:

| SETTING: outpatient, in patient, ED, home, nursing home, rehab, group, etc. | Clínica ambulatoria de obstetricia y ginecología. |
| --- | --- |
| PATIENT PROFILE: Information about the “patient” that helps select an SP and helps the learner get an understanding of them as a person. SP will know more information about the patient than learner will ever ask but allows SP to portray a fully developed patient personality. If none of the items below are particulars for the case, please write “Any answer acceptable.” | |
| Age range | 36 años. |
| Religious/spiritual background | Cualquier respuesta es aceptable. |
| Sex (e.g. male, female, intersex, transwoman, transman) | Hembra. |
| Sexual orientation (e.g. heterosexual, lesbian, gay, bisexual, pansexual, queer, asexual) | Cualquier respuesta es aceptable. |
| Gender expression (e.g. man, woman, genderqueer) | Cualquier respuesta es aceptable. |
| Race and ethnicity (e.g. to promote educational diversity, we use a diverse pool of SPs.) | Persona hispana/latina. |
| Physical description (e.g. BMI, height range) | Cualquier respuesta es aceptable. |
| Physical limitations | Cualquier respuesta es aceptable. |
| Patient appearance (e.g. disheveled, hospital gown, business casual, casual) | Estás alerta y vestido con ropa informal para trabajar. |
| Moulage + location (e.g. none, bruises, scars, body piercing, tattoos) | Cualquier respuesta es aceptable. |
| Affect (e.g. pleasant, cooperative) | Cooperativa. |
| Family group (e.g. who is family, who they live with) | Vive con su hijo de 10 años. |
| Education | Licenciatura de enfermería. |
| Level of health literacy | Nivel de alfabetización en salud alto. |
| Employment, if any - present and past, noting any current stresses | Trabaja como enfermera y tiene una agenda muy ocupada entre los turnos en la clínica y el cuidado de su hijo. |
| Home/homeless - type of dwelling, number of stories, owned or rented | Vives en una casa unifamiliar que está alquilada. |
| Financial situation - any current stresses | Cualquier respuesta es aceptable. |
| Insurance status (e.g. un/under/insured, public/private, HMO/PPO) | Cualquier respuesta es aceptable. |
| Habits (i.e., diet, exercise, caffeine, smoking, alcohol, drugs) | Has sido vegetariano durante los últimos 10 años. No bebes alcohol, no fumas ni consumes drogas. Corres 3 kilómetros tres veces por semana. |
| Activities (i.e., hobbies, sports, clubs, friends) | Disfrutas pasar tiempo con su hijo y apoyar a su equipo de fútbol. |
| Typical day - what is the usual daily routine | Te levantas temprano y preparas el desayuno y el almuerzo antes de ir a trabajar. Trabajas entre las 7:00 am y las 3:00 pm la mayoría de los días. Después del trabajo, recoges a su hijo de la escuela y lo llevas a la práctica de fútbol. Durante la práctica, sales a correr por el vecindario. Por las noches, te gusta leer o mirar televisión antes de irte a dormir. |

| CASE INFORMATION | |
| --- | --- |
| Chief Concern: What the patient will say when greeted by the student. The patient’s primary reason for seeking medical care often stated in their own words. | “No he tenido mi período en 3 meses.” |
| Additional Concerns: Other, if any, concerns the patient has today (i.e., symptoms, requests, expectations, etc.) that will become part of set agenda. | Ninguna. |
| THE PATIENT’S STORY: The SP will be asked to tell their symptom story and the personal and emotional impact for each of their concerns. You will want to write this in the patient’s voice. The symptom story should be able to answer this question: “Tell me more about [chief concern/additional concern], starting at the beginning and bringing me up to now.”  The personal context should be able to answer questions concerning the broader personal/psychosocial context of symptoms, especially the patient’s beliefs/attributions.  The emotional context should be able to ask how are you doing with this, how does this make you feel, how has this affected you emotionally? IMPACT: How has this affected your life? How has this been for your family? | “No me ha venido la regla en los últimos tres meses. Antes me venía la regla todos los meses, pero desde hace un año, mis ciclos se han vuelto menos frecuentes y ahora han cesado. Antes mi flujo era moderado, recuerdo que cambiaba mi compresa dos o tres veces al día, pero poco a poco se fueron haciendo mucho más ligeros hasta el punto de que solo usaba una compresa al día y ahora han cesado por completo. Llevo un estilo de vida bastante saludable y no sé a qué se deben estos cambios. ¿Podría estar pasando por una menopausia precoz?” |
| HISTORY OF PRESENT ILLNESS: Although some of the HPI will be given in the patient’s symptom story, the learners will expand the story during the direct question section. Below, describe the detailed history, usually about the chief concern, which the student must develop in order to make a useful assessment of the problem: | |
| Onset (when; gradual or sudden) | Hace 1 año; gradual. |
| Setting (what was going on or where was patient when symptoms first noticed?) | Alteraciones del ciclo menstrual. |
| Duration (how long) | 1 año hasta ahora. |
| Time relationships (frequency, constant or intermittent) | Los períodos menstruales (cada 4 semanas y con una duración de 4 a 5 días) han disminuido lentamente en frecuencia (cada 5 a 6 semanas) y han aumentado en duración (7 días). Ahora han cesado por completo. |
| Location | N/A |
| Radiation | N/A |
| Quality | N/A |
| Amount | El flujo menstrual ha disminuido de moderado (2-3 toallas sanitarias al día) a escaso (1 toalla sanitaria al día) o incluso a nada (0 toallas sanitarias al día). |
| Aggravated by what | N/A |
| Relieved by what | N/A |
| Associated with what | N/A |
| Attitude (what does the patient think is the problem, and how do they feel about it) | Has tenido ciclos menstruales regulares toda su vida y estás preocupada por estos cambios. Temes que puedas estar atravesando una menopausia de inicio temprano. |
| Overall course | El ciclo menstrual ha cesado lentamente. |
| REVIEW OF SYSTEMS: Significant positives and negatives | |
| NEGATIVES | POSITIVES |
| No ha notado cansancio, cambios en el estado de ánimo, bochornos, resequedad vaginal ni cambios urinarios. | Trastornos menstruales. |
| No ha tenido intolerancia al frío, cambios de voz, distintos hábitos intestinales. | Ha notado un incremento en el apetito y ha subido 15 libras en el último año. |
| No hay cambios visuales. | Ha notado algunos vellos de barba en mi barbilla y secreción de leche del pecho. |
|  |  |
| Past medical history |  |
| Medication allergies (name and reaction) | Ninguna. |
| Environmental allergies (name and reaction) | Ninguna. |
| Illnesses | Ninguna. |
| Vaccinations | Al día con todas las vacunas. |
| Surgeries | Ninguna. |
| Accidents/injuries/trauma | Ninguna. |
| Hospitalization | Parto de hijo. |
|  | |
| Inclusive sexual and reproductive history | |
| Sexual practices  Sexual partners  Protection: Use of safer sex practices  Use of birth control if appropriate  Risk of intimate partner violence | Cualquier respuesta es aceptable.  Usa anticonceptivos orales. |
| OB/GYN history | G1P1, embarazo normal a los 26 años con parto vaginal. Primera menstruación a los 14 años. El último Papanicolau fue hace 10 meses y se reportó normal. |
| Medications | Pastillas anticonceptivas una al día. |
| Immunizations | - Tétano - Gripe - Hepatitis - Vacuna antineumocócica - VPH - Otros: COVID |
| Tobacco products   - Cigarrillos - Puros - Pipas - Masticables - Cigarrillos electrónicos | - Nunca - Pasado – año de inicio/año de abandono - Actual   - Cantidad   - # de años |
| Alcohol   - Cerveza - Vino - Licor - Otros | - Nunca - Pasado – año de inicio/año de abandono - Actual   - Cantidad   - # de años |
| Drugs   - Marihuana - Cocaína - Heroína - Metanfetamina - Drogas intravenosas - Inhalantes - Otros | - Nunca - Pasado – año de inicio/año de abandono - Actual   - Cantidad   - # de años |
| Diet (describe) | Has sido vegetariano durante los últimos 10 años. |
| Exercise (describe) | Corres 3 kilómetros tres veces por semana. |
| List any other important social history or information important to this case | Ninguna. |
| Family history |  |
| Mother, father, siblings, grandparents, and other significant findings | Sus padres son sanos y están vivos. Su madre tuvo la menopausia a los 55 años. |
|  |  |
| Physical Exam - List exam maneuvers expected for this case and any abnormal findings that SP will simulate. (tenderness, hyper-hypo reflex, rebound, weakness, etc.)  María aparecerá alerta y comprometida durante todo el encuentro.  No se realizará ningún examen físico durante este caso. | |
| PHYSICAL EXAM FINDINGS |  |
| 1. Written in layperson’s terms |  |
| 1. General appearance - affect, appearance, position of patient at opening (i.e., sitting, lying down, holding abdomen, etc.) | Cuando el estudiante se una a la videollamada deberá estar sentada en una silla vistienda su ropa habitual. |
| 1. Vital signs | Temperatura: 98.5° F  Pulso: 75 bpm  Presión arterial: 118/64  Frecuencia respiratoria: 14 |
| 1. Specific findings and affect | María aparecerá alerta y comprometida durante todo el encuentro. |
| 1. Response to certain physical movements | N/A |
|  |  |
| DIAGNOSIS AND DIFFERENTIAL |  |
| Diagnosis with support from positive and negative history and PE findings | Síndrome de Ovario Poliquístico |
| Differential with support from positive and negative history and PE findings | Hiperprolactinemia, Hipotiroidismo, Insuficiencia ovárica prematura (menopausia precoz), Causas secundarias de amenorrea (estrés, ejercicio excesivo). |
|  |  |
| MANAGEMENT OR DIAGNOSTIC PLAN | Asegurarle a la paciente que comprendemos sus preocupaciones y explicarle que realizaremos una evaluación diagnóstica exhaustiva. Esto incluirá pruebas para evaluar las posibles causas hormonales, como la insuficiencia ovárica prematura, y una ecografía pélvica. |
|  |  |
| PROFESSIONALISM ISSUES OR CHALLENGES | Competencia cultural. |
