# Supplementary material for: A Standardized Clinical Case-Based Assessment for Evaluating Medical Students' Oral Spanish Communication Skills
Source: MedEdPORTAL. 2025 Apr 17;21:11518. doi: 10.15766/mep_2374-8265.11518 (PMC12003672; doi:10.15766/mep_2374-8265.11518)
Supplement: Supplementary file 1 — Precourse Self-Assessment Video.mp4Patient-Provider Interaction Checklist.docxSP Case Spanish.docxSP Case English.docxSP Pilot Case 1 Spanish.docxSP Pilot Case 1 English.docxSP Pilot Case 2 Spanish.docxSP Pilot Case 2 English.docxSP Pilot Case 3 Spanish.docxSP Pilot Case 3 English.docxFacilitators Guide.docx [file mep_2374-8265.11518-s001.zip › F. SP Pilot Case 1 English.docx]

Appendix F: Standardized Patient Case Development Tool Pilot Case 1 English

Instructions: Facilitator and Standardized Patient should use the Standardized Patient script to conduct the student communication skills assessment

Primary Case Author: Cristina Aguayo-Mazzucato, MD PhD

Secondary Case Author: Brandon Martel

Name of Case: Menstrual Problems

Name of Educational and/or Assessment Activity: Pilot Case for Medical Spanish

Type and Level of Learner: Intermediate to Advanced Medical Spanish Student

Patient Name: María Cortinez

Chief Concern: No periods

Most Likely Diagnosis and Differential with Rationale from History and/or Physical Exam: The most likely diagnosis is Polycystic Ovary Syndrome (PCOS). The patient presents with secondary amenorrhea, weight gain, hirsutism (facial hair on the chin), and possibly galactorrhea (breast milk secretion). These symptoms are consistent with PCOS, a common endocrine disorder that can cause irregular menstrual cycles and elevated androgen levels. Other conditions in the differential diagnosis include: Hyperprolactinemia, possibly caused by a pituitary adenoma (prolactinoma) or other hormonal imbalances. Hypothyroidism can lead to amenorrhea, galactorrhea, and weight gain. Abnormal thyroid function can affect menstrual cycles and metabolism, explaining some of the patient’s symptoms. Premature ovarian insufficiency (early menopause), which could be related to genetic or autoimmune factors. Secondary causes of amenorrhea (such as stress or excessive exercise). Although the patient leads a healthy lifestyle, work-related stress, and physical activity (running several times a week) could be contributing to her menstrual irregularities. However, symptoms like galactorrhea suggest a more hormonal cause.

Domains: Check all that apply

- Professionalism
- Communication and Interpersonal Skills
- Medical History
- Physical Exam
- Shared Decision-Making
- Patient Education
- Clinical Reasoning
- Documentation
- Handoff
- Presentation
- Other:

Case Objectives: Please list specific objectives for each of the domains you have checked above

1. Exhibit cultural competence by being sensitive to the patient's background and lifestyle choices, such as her vegetarian diet and exercise habits, without making assumptions.
2. Build rapport with the patient by actively listening, addressing her concerns about menopause and hormonal imbalances, and creating a comfortable environment for open communication.
3. Gather a comprehensive medical history focused on the patient's menstrual cycle, hormonal changes, and lifestyle factors, paying special attention to any family history of endocrine or reproductive issues.

Standardized Patient Script:

| SETTING: outpatient, in patient, ED, home, nursing home, rehab, group, etc. | OB-GYN outpatient clinic. |
| --- | --- |
| PATIENT PROFILE: Information about the “patient” that helps select an SP and helps the learner get an understanding of them as a person. SP will know more information about the patient than learner will ever ask but allows SP to portray a fully developed patient personality. If none of the items below are particulars for the case, please write “Any answer acceptable.” | |
| Age range | 36 years old. |
| Religious/spiritual background | Any answer acceptable. |
| Sex (e.g. male, female, intersex, transwoman, transman) | Female. |
| Sexual orientation (e.g. heterosexual, lesbian, gay, bisexual, pansexual, queer, asexual) | Any answer acceptable. |
| Gender expression (e.g. man, woman, genderqueer) | Any answer acceptable. |
| Race and ethnicity (e.g. to promote educational diversity, we use a diverse pool of SPs.) | Hispanic/Latinx. |
| Physical description (e.g. BMI, height range) | Any answer acceptable. |
| Physical limitations | Any answer acceptable. |
| Patient appearance (e.g. disheveled, hospital gown, business casual, casual) | You are alert and dressed in casual clothing for work. |
| Moulage + location (e.g. none, bruises, scars, body piercing, tattoos) | Any answer acceptable. |
| Affect (e.g. pleasant, cooperative) | Cooperative. |
| Family group (e.g. who is family, who they live with) | You live with your 10-year-old son. |
| Education | Nursing degree. |
| Level of health literacy | High health literacy. |
| Employment, if any - present and past, noting any current stresses | You work as a nurse and have a busy schedule between clinic shifts and taking care of your son. |
| Home/homeless - type of dwelling, number of stories, owned or rented | You live in a single unit home that is rented. |
| Financial situation - any current stresses | Any answer acceptable. |
| Insurance status (e.g. un/under/insured, public/private, HMO/PPO) | Any answer acceptable. |
| Habits (i.e., diet, exercise, caffeine, smoking, alcohol, drugs) | You have been a vegetarian during the last 10 years. You do not drink alcohol, smoke or consume drugs. You run 2 miles three times per week. |
| Activities (i.e., hobbies, sports, clubs, friends) | You enjoy spending time with your son and supporting his soccer team. |
| Typical day - what is the usual daily routine | You rise early and prepare breakfast and lunch before going to work. You work between 7am and 3pm most days. After work, you pick up your son from school and drive him to soccer practice. During practice, you go on a run through the neighborhood. In the evenings, you like to read or watch TV before going to bed. |

| CASE INFORMATION | |
| --- | --- |
| Chief Concern: What the patient will say when greeted by the student. The patient’s primary reason for seeking medical care often stated in their own words. | “I haven’t had my period in 3 months.” |
| Additional Concerns: Other, if any, concerns the patient has today (i.e., symptoms, requests, expectations, etc.) that will become part of set agenda. | None. |
| THE PATIENT’S STORY: The SP will be asked to tell their symptom story and the personal and emotional impact for each of their concerns. You will want to write this in the patient’s voice. The symptom story should be able to answer this question: “Tell me more about [chief concern/additional concern], starting at the beginning and bringing me up to now.”  The personal context should be able to answer questions concerning the broader personal/psychosocial context of symptoms, especially the patient’s beliefs/attributions.  The emotional context should be able to ask how are you doing with this, how does this make you feel, how has this affected you emotionally? IMPACT: How has this affected your life? How has this been for your family? | “I haven’t had my period in the last three months. Before, I used to get my period every month. But for the past year, my cycles have become less frequent and now have stopped. My flow used to be moderate, I remember changing my pad 2-3 times per day. But slowly, they became much lighter to the point that I would only use one pad per day and now they have stopped all together. I lead a fairly healthy lifestyle and don’t know what these changes are caused by. Could I be going through early menopause?” |
| HISTORY OF PRESENT ILLNESS: Although some of the HPI will be given in the patient’s symptom story, the learners will expand the story during the direct question section. Below, describe the detailed history, usually about the chief concern, which the student must develop in order to make a useful assessment of the problem: | |
| Onset (when; gradual or sudden) | 1 year ago; gradual. |
| Setting (what was going on or where was patient when symptoms first noticed?) | Disturbances of menstrual cycle. |
| Duration (how long) | 1 year so far. |
| Time relationships (frequency, constant or intermittent) | Monthly periods (every 4 weeks lasting 4-5 days) have slowly decreased in frequency (every 5-6 weeks) and increased in duration (7 days). Now they have completely stopped. |
| Location | N/A |
| Radiation | N/A |
| Quality | N/A |
| Amount | Period flow has decreased from moderate (2-3 pads daily) to scant (1 pad daily) to nothing (0 pads daily). |
| Aggravated by what | N/A |
| Relieved by what | N/A |
| Associated with what | N/A |
| Attitude (what does the patient think is the problem, and how do they feel about it) | You have had regular menstrual cycles your whole life and are worried by these changes. You are concerned that you might be going through early onset menopause. |
| Overall course | Menstrual cycle has slowly ceased. |
| REVIEW OF SYSTEMS: Significant positives and negatives | |
| NEGATIVES | POSITIVES |
| No fatigue, mood changes, hot flashes, vaginal dryness or urinary changes. | Menstrual disturbances. |
| Denies cold intolerance, voice changes, alteration in bowel movements. | 15lb weight gain and increased hunger over past year |
| No visual changes. | Growth of thick hairs on chin and milk secretion from breasts. |
|  |  |
| Past medical history |  |
| Medication allergies (name and reaction) | None. |
| Environmental allergies (name and reaction) | None. |
| Illnesses | None. |
| Vaccinations | Up to date with all vaccines. |
| Surgeries | None. |
| Accidents/injuries/trauma | None. |
| Hospitalization | Delivery of son. |
|  | |
| Inclusive sexual and reproductive history | |
| Sexual practices  Sexual partners  Protection: Use of safer sex practices  Use of birth control if appropriate  Risk of intimate partner violence | Any answer acceptable.  Uses oral contraception. |
| OB/GYN history | G1P1, normal pregnancy at age 26 with vaginal delivery. First menses at age 14. Last Pap smear was 10 months ago and normal. |
| Medications | Oral contraceptives once a day. |
| Immunizations | - Tetanus - Flu - Hepatitis - Pneumovax - HPV - Other: COVID |
| Tobacco products   - Cigarettes - Cigar - Pipe - Chew - E-cigarettes | - Never - Past - year started/year quit - Current   - Quantity   - # of years |
| Alcohol   - Beer - Wine - Liquor - Other | - Never - Past - year started/year quit - Current   - 2 bottles on weekends   - 35 years |
| Drugs   - Weed - Cocaine - Heroin - Meth - IV - Inhalants - Other | - Never - Past - year started/year quit - Current   - Quantity   - # of years |
| Diet (describe) | You have been a vegetarian during the last 10 years. |
| Exercise (describe) | You run 2 miles three times per week. |
| List any other important social history or information important to this case | None. |
| Family history |  |
| Mother, father, siblings, grandparents, and other significant findings | Your parents are alive and healthy. Your mother had menopause at 55 years of age. |
|  |  |
| Physical Exam - List exam maneuvers expected for this case and any abnormal findings that SP will simulate. (tenderness, hyper-hypo reflex, rebound, weakness, etc.)  María will appear alert and engaged throughout the encounter.  There is no physical examination during this case. | |
| PHYSICAL EXAM FINDINGS |  |
| 1. Written in layperson’s terms |  |
| 1. General appearance - affect, appearance, position of patient at opening (i.e., sitting, lying down, holding abdomen, etc.) | When the student joins the video call you should be sitting in a chair wearing your regular clothes. |
| 1. Vital signs | T: 98.5° F  Pulse: 75 bpm  BP: 118/64  RR: 14 |
| 1. Specific findings and affect | María will appear alert and engaged throughout the encounter. |
| 1. Response to certain physical movements | N/A |
|  |  |
| DIAGNOSIS AND DIFFERENTIAL |  |
| Diagnosis with support from positive and negative history and PE findings | Polycystic Ovary Syndrome (PCOS) |
| Differential with support from positive and negative history and PE findings | Hyperprolactinemia, Hypothyroidism, Premature ovarian insufficiency (early menopause), Secondary causes of amenorrhea (stress, excessive exercise). |
|  |  |
| MANAGEMENT OR DIAGNOSTIC PLAN | Reassure the patient that we understand her concerns and explain that we will conduct a thorough diagnostic evaluation. This will include tests to assess possible hormonal causes, such as premature ovarian insufficiency, as well as a pelvic ultrasound. |
|  |  |
| PROFESSIONALISM ISSUES OR CHALLENGES | Cultural competency. |
