# Supplementary material for: A Standardized Clinical Case-Based Assessment for Evaluating Medical Students' Oral Spanish Communication Skills
Source: MedEdPORTAL. 2025 Apr 17;21:11518. doi: 10.15766/mep_2374-8265.11518 (PMC12003672; doi:10.15766/mep_2374-8265.11518)
Supplement: Supplementary file 1 — Precourse Self-Assessment Video.mp4Patient-Provider Interaction Checklist.docxSP Case Spanish.docxSP Case English.docxSP Pilot Case 1 Spanish.docxSP Pilot Case 1 English.docxSP Pilot Case 2 Spanish.docxSP Pilot Case 2 English.docxSP Pilot Case 3 Spanish.docxSP Pilot Case 3 English.docxFacilitators Guide.docx [file mep_2374-8265.11518-s001.zip › G. SP Pilot Case 2 Spanish.docx]

Appendix G: Standardized Patient Case Development Tool Pilot Case 2 Spanish

Instructions: Facilitator and Standardized Patient should use the Standardized Patient script to conduct the student communication skills assessment

Primary Case Author: Cristina Aguayo-Mazzucato, MD PhD

Secondary Case Author: Brandon Martel

Name of Case: Ictericia

Name of Educational and/or Assessment Activity: Caso piloto de español medico

Type and Level of Learner: Estudiante de español médico de nivel intermedio a avanzado

Patient Name: Fernanda López

Chief Concern: Ojos y piel amarillos

Most Likely Diagnosis and Differential with Rationale from History and/or Physical Exam: El diagnóstico más probable para esta paciente es hepatitis viral aguda (probablemente hepatitis A o hepatitis B). Los síntomas clave, como la ictericia (piel y ojos amarillos), heces de color claro, orina oscura, prurito severo (picazón) y dolor en el cuadrante superior derecho del abdomen, son consistentes con una hepatitis aguda. Además, la paciente menciona haber viajado a México recientemente sin vacunarse, lo que incrementa la probabilidad de una infección viral transmitida por agua o alimentos contaminados. Diagnóstico diferencial incluye: obstrucción de las vías biliares, posiblemente causada por cálculos biliares o estenosis biliar. Sin embargo, la paciente no menciona fiebre ni escalofríos, que son más característicos de una colangitis. Enfermedad hepática crónica, como la cirrosis biliar primaria que es una enfermedad autoimmune que afecta principalmente a mujeres de mediana edad, pero los síntomas agudos no son tan comunes como en la hepatitis viral. Hepatitis medicamentosa ya que la paciente menciona el uso de 4 pastillas diarias de Tylenol (acetaminofén), lo que podría causar daño hepático si se consume en dosis excesivas. Aunque esto podría explicar el dolor abdominal y la ictericia, la historia de un viaje reciente sugiere más una causa infecciosa. La historia de transfusión sanguínea hace 20 años podría aumentar el riesgo de hepatitis crónica no diagnosticada (como hepatitis C), que con el tiempo podría llevar a insuficiencia hepática. Sin embargo, los síntomas agudos sugieren más una hepatitis viral reciente.

Domains: Check all that apply

- Professionalism
- Communication and Interpersonal Skills
- Medical History
- Physical Exam
- Shared Decision-Making
- Patient Education
- Clinical Reasoning
- Documentation
- Handoff
- Presentation
- Other:

Case Objectives: Please list specific objectives for each of the domains you have checked above

1. Demuestre empatía y respeto al hablar de temas delicados, como los síntomas del paciente, los posibles diagnósticos y las inquietudes sobre su salud.
2. Establezca una comunicación clara y abierta con el paciente escuchando activamente y abordando sus inquietudes.
3. Obtenga un historial médico completo y detallado, centrándose en factores clave como el historial de viajes, los hábitos de estilo de vida, el uso de medicamentos y cualquier intervención médica previa relevante para los síntomas actuales.

Standardized Patient Script:

| SETTING: outpatient, in patient, ED, home, nursing home, rehab, group, etc. | Servicio de urgencias a las 22h. |
| --- | --- |
| PATIENT PROFILE: Information about the “patient” that helps select an SP and helps the learner get an understanding of them as a person. SP will know more information about the patient than learner will ever ask but allows SP to portray a fully developed patient personality. If none of the items below are particulars for the case, please write “Any answer acceptable.” | |
| Age range | 52 años. |
| Religious/spiritual background | Cualquier respuesta es aceptable. |
| Sex (e.g. male, female, intersex, transwoman, transman) | Hembra. |
| Sexual orientation (e.g. heterosexual, lesbian, gay, bisexual, pansexual, queer, asexual) | Cualquier respuesta es aceptable. |
| Gender expression (e.g. man, woman, genderqueer) | Cualquier respuesta es aceptable. |
| Race and ethnicity (e.g. to promote educational diversity, we use a diverse pool of SPs.) | Persona hispana/latina. |
| Physical description (e.g. BMI, height range) | Cualquier respuesta es aceptable. |
| Physical limitations | Cualquier respuesta es aceptable. |
| Patient appearance (e.g. disheveled, hospital gown, business casual, casual) | Llevas pijama y abrigo. |
| Moulage + location (e.g. none, bruises, scars, body piercing, tattoos) | Cualquier respuesta es aceptable. |
| Affect (e.g. pleasant, cooperative) | Eres visiblemente incómoda. |
| Family group (e.g. who is family, who they live with) | Cualquier respuesta es aceptable. |
| Education | Título universitario. |
| Level of health literacy | Nivel de alfabetización en salud intermedio. |
| Employment, if any - present and past, noting any current stresses | Usted es agente de viajes. |
| Home/homeless - type of dwelling, number of stories, owned or rented | Cualquier respuesta es aceptable. |
| Financial situation - any current stresses | Cualquier respuesta es aceptable. |
| Insurance status (e.g. un/under/insured, public/private, HMO/PPO) | Cualquier respuesta es aceptable. |
| Habits (i.e., diet, exercise, caffeine, smoking, alcohol, drugs) | Toma dos copas de vino al día durante los últimos 30 años. |
| Activities (i.e., hobbies, sports, clubs, friends) | Te gusta viajar y fuiste a México hace dos meses. |
| Typical day - what is the usual daily routine | Cualquier respuesta es aceptable. |

| CASE INFORMATION | |
| --- | --- |
| Chief Concern: What the patient will say when greeted by the student. The patient’s primary reason for seeking medical care often stated in their own words. | “Soy tan amarillo que parezco un limón.” |
| Additional Concerns: Other, if any, concerns the patient has today (i.e., symptoms, requests, expectations, etc.) that will become part of set agenda. | Ninguna. |
| THE PATIENT’S STORY: The SP will be asked to tell their symptom story and the personal and emotional impact for each of their concerns. You will want to write this in the patient’s voice. The symptom story should be able to answer this question: “Tell me more about [chief concern/additional concern], starting at the beginning and bringing me up to now.”  The personal context should be able to answer questions concerning the broader personal/psychosocial context of symptoms, especially the patient’s beliefs/attributions.  The emotional context should be able to ask how are you doing with this, how does this make you feel, how has this affected you emotionally? IMPACT: How has this affected your life? How has this been for your family? | “Comenzó hace tres semanas cuando mis heces se volvieron blancas y mi orina muy oscura. Unos días después, mi piel y mis ojos comenzaron a ponerse amarillos. ¡Me pica muchísimo la piel! Benadryl me ayudó al principio, pero ya no. Además de estos cambios, también he tenido dolor abdominal y náuseas durante las últimas 3 semanas. El dolor ocurre una vez al día en esta zona (el paciente señala el cuadrante superior derecho del abdomen) y mejora con Tylenol. Nunca antes había tenido un dolor como este ni estos cambios de color. ¿Qué me está pasando?” |
| HISTORY OF PRESENT ILLNESS: Although some of the HPI will be given in the patient’s symptom story, the learners will expand the story during the direct question section. Below, describe the detailed history, usually about the chief concern, which the student must develop in order to make a useful assessment of the problem: | |
| Onset (when; gradual or sudden) | Hace 3 semanas; inicio rápido. |
| Setting (what was going on or where was patient when symptoms first noticed?) | Habías visitado México hace 2 meses y no se puso ninguna vacuna antes de viajar. |
| Duration (how long) | 3 semanas hasta ahora. |
| Time relationships (frequency, constant or intermittent) | Picazón constante; cambios continuos de color de las deposiciones/orina/piel/ojos; dolor abdominal intermitente y náuseas una vez al día. |
| Location | Coloración amarillenta de la piel y los ojos; dolor abdominal en el cuadrante superior derecho. |
| Radiation | El dolor abdominal no se transmite a otras partes del cuerpo. |
| Quality | El dolor abdominal es sordo. |
| Amount | Califica su picazón con un 7 sobre 10 y su dolor abdominal con un 3 sobre 10. |
| Aggravated by what | No hay nada que aumente el dolor abdominal. |
| Relieved by what | La picazón se alivió inicialmente con Benadryl, pero ya no; el dolor abdominal mejora tomando 4 pastillas de Tylenol al día. |
| Associated with what | El dolor abdominal se asocia a náuseas; las comidas o el ayuno no tienen ningún impacto sobre el dolor. |
| Attitude (what does the patient think is the problem, and how do they feel about it) | Estás preocupado por estos cambios. Te avergüenza el color amarillento de su piel y deseas desesperadamente aliviar la picazón. |
| Overall course | Picazón constante y decoloración de heces/orina/piel/ojos; dolor abdominal intermitente y náuseas. |
| REVIEW OF SYSTEMS: Significant positives and negatives | |
| NEGATIVES | POSITIVES |
| No ha tenido cambios en los hábitos intestinales (aparte del color de las heces y la orina). | Heces blancas, orina muy oscura, piel y ojos amarillentos. |
| Niega fiebre y sudores nocturnos, cambios de peso, dolor de articulaciones. | Picazón en la piel. |
| Sin vomitar. | Dolor abdominal, náuseas, pérdida del apetito, y mucha fatiga. |
|  |  |
| Past medical history |  |
| Medication allergies (name and reaction) | Penicilina. |
| Environmental allergies (name and reaction) | Ninguna. |
| Illnesses | Hipotiroidismo durante los últimos 5 años. |
| Vaccinations | No está al día con las vacunas. Nunca te han hecho una colonoscopia. |
| Surgeries | Tuviste dos cesáreas cuando tenías 25 y 32 años. Recibió una transfusión sanguínea hace 20 años durante la última cesárea. A los 35 años le ligaron las trompas de Falopio. |
| Accidents/injuries/trauma | Ninguna. |
| Hospitalization | Partos a los 25 y 32 años. |
|  | |
| Inclusive sexual and reproductive history | |
| Sexual practices  Sexual partners  Protection: Use of safer sex practices  Use of birth control if appropriate  Risk of intimate partner violence | Cualquier respuesta es aceptable. |
| OB/GYN history | G2P2, embarazos normales con partos por cesárea a los 25 y 32 años. A los 35 años te realizaron una ligadura de trompas. |
| Medications | Tylenol 500 mg x 4 pastillas al día  Hormonas tiroideas diarias |
| Immunizations | - Tétano - Gripe - Hepatitis - Vacuna antineumocócica - VPH - Otros: COVID |
| Tobacco products   - Cigarrillos - Puros - Pipas - Masticables - Cigarrillos electrónicos | - Nunca - Pasado – año de inicio/año de abandono - Actual   - Cantidad   - # de años |
| Alcohol   - Cerveza - Vino - Licor - Otros | - Nunca - Pasado – año de inicio/año de abandono Past - year started/year quit - Actual   - 2 copas de vino al día   - 30 años |
| Drugs   - Marihuana - Cocaína - Heroína - Metanfetamina - Drogas intravenosas - Inhalantes - Otros | - Nunca - Pasado – año de inicio/año de abandono - Actual   - Cantidad   - # de años |
| Diet (describe) | Cualquier respuesta es aceptable. |
| Exercise (describe) | Cualquier respuesta es aceptable. |
| List any other important social history or information important to this case | Ninguna. |
| Family history |  |
| Mother, father, siblings, grandparents, and other significant findings | Su padre murió a los 55 años de cáncer pancreático. Su madre está viva y es sana. |
|  |  |
| Physical Exam - List exam maneuvers expected for this case and any abnormal findings that SP will simulate. (tenderness, hyper-hypo reflex, rebound, weakness, etc.)  Fernanda se mostrará visiblemente incómoda durante el encuentro. Ocasionalmente se rascará la piel durante la visita y se sujetará la parte superior derecha del abdomen para expresar dolor.  No se realizará ningún examen físico durante este caso. | |
| PHYSICAL EXAM FINDINGS |  |
| 1. Written in layperson’s terms |  |
| 1. General appearance - affect, appearance, position of patient at opening (i.e., sitting, lying down, holding abdomen, etc.) | Cuando el estudiante se una a la videollamada deberá estar sentado en una silla vistiendo su pijama y un abrigo (o su ropa habitual). |
| 1. Vital signs | Temperatura: 98.2° F  Pulso: 75 bpm  Presión arterial: 125/64  Frecuencia respiratoria: 22 |
| 1. Specific findings and affect | Fernanda se mostrará visiblemente incómoda durante el encuentro. |
| 1. Response to certain physical movements | Fernanda se rascará la piel durante la visita y se sujetará la parte superior derecha del abdomen para expresar dolor. |
|  |  |
| DIAGNOSIS AND DIFFERENTIAL |  |
| Diagnosis with support from positive and negative history and PE findings | Hepatitis viral aguda (probablemente hepatitis A) |
| Differential with support from positive and negative history and PE findings | Cálculos biliares o estenosis biliar, cirrosis biliar primaria, hepatitis medicamentosa, hepatitis crónica no diagnosticada (como hepatitis C). |
|  |  |
| MANAGEMENT OR DIAGNOSTIC PLAN | Explicar las posibles causas, mencionando que se realizará una evaluación diagnóstica que incluirá pruebas serológicas para hepatitis A, B y C, pruebas de función hepática y una ecografía abdominal para evaluar los conductos biliares y descartar obstrucción. Si se confirma hepatitis viral, se procederá con un manejo de soporte, incluyendo hidratación y monitoreo de la función hepática. |
|  |  |
| PROFESSIONALISM ISSUES OR CHALLENGES | Competencia cultural. |
