# Supplementary material for: A Standardized Clinical Case-Based Assessment for Evaluating Medical Students' Oral Spanish Communication Skills
Source: MedEdPORTAL. 2025 Apr 17;21:11518. doi: 10.15766/mep_2374-8265.11518 (PMC12003672; doi:10.15766/mep_2374-8265.11518)
Supplement: Supplementary file 1 — Precourse Self-Assessment Video.mp4Patient-Provider Interaction Checklist.docxSP Case Spanish.docxSP Case English.docxSP Pilot Case 1 Spanish.docxSP Pilot Case 1 English.docxSP Pilot Case 2 Spanish.docxSP Pilot Case 2 English.docxSP Pilot Case 3 Spanish.docxSP Pilot Case 3 English.docxFacilitators Guide.docx [file mep_2374-8265.11518-s001.zip › H. SP Pilot Case 2 English.docx]

Appendix H: Standardized Patient Case Development Tool Pilot Case 2 English

Instructions: Facilitator and Standardized Patient should use the Standardized Patient script to conduct the student communication skills assessment

Primary Case Author: Cristina Aguayo-Mazzucato, MD PhD

Secondary Case Author: Brandon Martel

Name of Case: Jaundice

Name of Educational and/or Assessment Activity: Pilot Case for Medical Spanish

Type and Level of Learner: Intermediate to Advanced Medical Spanish Student

Patient Name: Fernanda López

Chief Concern: yellow eyes and skin

Most Likely Diagnosis and Differential with Rationale from History and/or Physical Exam: The most likely diagnosis for this patient is acute viral hepatitis (probably hepatitis A or hepatitis B). Key symptoms, such as jaundice (yellowing of the skin and eyes), light-colored stools, dark urine, severe pruritus (itching), and pain in the right upper quadrant of the abdomen, are consistent with acute hepatitis. The patient mentions having recently traveled to Mexico without getting vaccinated, which increases the likelihood of a viral infection transmitted through contaminated water or food. The differential diagnosis includes biliary obstruction, possibly caused by gallstones or biliary stenosis. However, the patient does not mention fever or chills, which are more characteristic of cholangitis. Chronic liver disease, such as primary biliary cirrhosis, an autoimmune disease that primarily affects middle-aged women, could also be considered, however acute symptoms are less common compared to viral hepatitis. Another possibility is drug-induced hepatitis, as the patient reports taking 4 Tylenol (acetaminophen) pills daily, which could cause liver damage if consumed in excessive doses. While this could explain the abdominal pain and jaundice, the recent travel history points more towards an infectious cause. Finally, the history of a blood transfusion 20 years ago could increase the risk of undiagnosed chronic hepatitis (such as hepatitis C), which over time could lead to liver failure. However, the acute symptoms suggest a more recent viral hepatitis infection.

Domains: Check all that apply

- Professionalism
- Communication and Interpersonal Skills
- Medical History
- Physical Exam
- Shared Decision-Making
- Patient Education
- Clinical Reasoning
- Documentation
- Handoff
- Presentation
- Other:

Case Objectives: Please list specific objectives for each of the domains you have checked above

1. Demonstrate empathy and respect when discussing sensitive topics such as the patient's symptoms, possible diagnoses, and concerns about their health.
2. Establish clear and open communication with the patient, actively listening and addressing their concerns.
3. Obtain a complete and detailed medical history, focusing on key factors such as travel history, lifestyle habits, medication use, and any previous medical interventions relevant to the current symptoms.

Standardized Patient Script:

| SETTING: outpatient, in patient, ED, home, nursing home, rehab, group, etc. | Emergency department at 10pm. |
| --- | --- |
| PATIENT PROFILE: Information about the “patient” that helps select an SP and helps the learner get an understanding of them as a person. SP will know more information about the patient than learner will ever ask but allows SP to portray a fully developed patient personality. If none of the items below are particulars for the case, please write “Any answer acceptable.” | |
| Age range | 52 years old. |
| Religious/spiritual background | Any answer acceptable. |
| Sex (e.g. male, female, intersex, transwoman, transman) | Female. |
| Sexual orientation (e.g. heterosexual, lesbian, gay, bisexual, pansexual, queer, asexual) | Any answer acceptable. |
| Gender expression (e.g. man, woman, genderqueer) | Any answer acceptable. |
| Race and ethnicity (e.g. to promote educational diversity, we use a diverse pool of SPs.) | Hispanic/Latinx. |
| Physical description (e.g. BMI, height range) | Any answer acceptable. |
| Physical limitations | Any answer acceptable. |
| Patient appearance (e.g. disheveled, hospital gown, business casual, casual) | Dressed in pajamas and a coat. |
| Moulage + location (e.g. none, bruises, scars, body piercing, tattoos) | Any answer acceptable. |
| Affect (e.g. pleasant, cooperative) | Visibly uncomfortable. |
| Family group (e.g. who is family, who they live with) | Any answer acceptable. |
| Education | College degree. |
| Level of health literacy | Intermediate health literacy. |
| Employment, if any - present and past, noting any current stresses | You work as a travel agent. |
| Home/homeless - type of dwelling, number of stories, owned or rented | Any answer acceptable. |
| Financial situation - any current stresses | Any answer acceptable. |
| Insurance status (e.g. un/under/insured, public/private, HMO/PPO) | Any answer acceptable. |
| Habits (i.e., diet, exercise, caffeine, smoking, alcohol, drugs) | You drink two glasses of wine every day for the last 30 years. |
| Activities (i.e., hobbies, sports, clubs, friends) | You enjoy travel and went to Mexico two months ago. |
| Typical day - what is the usual daily routine | Any answer acceptable. |

| CASE INFORMATION | |
| --- | --- |
| Chief Concern: What the patient will say when greeted by the student. The patient’s primary reason for seeking medical care often stated in their own words. | “I’m so yellow I look like a lemon.” |
| Additional Concerns: Other, if any, concerns the patient has today (i.e., symptoms, requests, expectations, etc.) that will become part of set agenda. | None. |
| THE PATIENT’S STORY: The SP will be asked to tell their symptom story and the personal and emotional impact for each of their concerns. You will want to write this in the patient’s voice. The symptom story should be able to answer this question: “Tell me more about [chief concern/additional concern], starting at the beginning and bringing me up to now.”  The personal context should be able to answer questions concerning the broader personal/psychosocial context of symptoms, especially the patient’s beliefs/attributions.  The emotional context should be able to ask how are you doing with this, how does this make you feel, how has this affected you emotionally? IMPACT: How has this affected your life? How has this been for your family? | “It started three weeks ago when my bowel movements turned white and my urine became very dark. A few days later, my skin and eyes started turning yellow. My skin is extremely itchy! Benadryl helped at first but not anymore. On top of these changes, I have had abdominal pain and nausea for the past 3 weeks too. The pain occurs once a day in this area (patient points to right upper quadrant of abdomen) and improves with Tylenol. I have never had a pain like this before, or these color changes. What is happening to me?” |
| HISTORY OF PRESENT ILLNESS: Although some of the HPI will be given in the patient’s symptom story, the learners will expand the story during the direct question section. Below, describe the detailed history, usually about the chief concern, which the student must develop in order to make a useful assessment of the problem: | |
| Onset (when; gradual or sudden) | 3 weeks ago; rapid onset. |
| Setting (what was going on or where was patient when symptoms first noticed?) | You had visited Mexico 2 months ago. You did not receive any vaccines before going. |
| Duration (how long) | 3 weeks so far. |
| Time relationships (frequency, constant or intermittent) | Constant itchiness; ongoing color changes of bowel movements/urine/skin/eyes; intermittent abdominal pain and nausea once a day. |
| Location | Yellowing of skin and eyes; abdominal pain in right upper quadrant. |
| Radiation | Abdominal pain does not travel to other parts of the body. |
| Quality | Abdominal pain is dull. |
| Amount | You rate your itchiness as a 7 out of 10 and your abdominal pain as a 3 out of 10. |
| Aggravated by what | There is nothing that increases the abdominal pain. |
| Relieved by what | The itchiness was initially relieved by Benadryl but not anymore; the abdominal pain is improved by taking 4 Tylenol pills a day. |
| Associated with what | The abdominal pain is associated with nausea; meals or fasting have no impact on the pain. |
| Attitude (what does the patient think is the problem, and how do they feel about it) | You are worried about these changes. You are embarrassed by the yellowing of your skin and desperately want relief from the itchiness. |
| Overall course | Constant itchiness and discoloration of stool/urine/skin/eyes; intermittent abdominal pain and nausea. |
| REVIEW OF SYSTEMS: Significant positives and negatives | |
| NEGATIVES | POSITIVES |
| No changes in bowel movements or urination (apart from color). | White stool, very dark urine, yellowed skin and eyes. |
| No fever, night sweats, weight changes, or joint pain. | Itchy skin. |
| No vomiting. | Abdominal pain, nausea, loss of appetite, and severe fatigue. |
|  |  |
| Past medical history |  |
| Medication allergies (name and reaction) | Penicillin. |
| Environmental allergies (name and reaction) | None. |
| Illnesses | Hypothyroidism for the past 5 years. |
| Vaccinations | Not up to date with vaccines. You have never had a colonoscopy. |
| Surgeries | You had 2 C-sections when you were 25 and 32 years old. You got a blood transfusion 20 years ago during the last C-section. At 35 years old you had a tubal ligation. |
| Accidents/injuries/trauma | None. |
| Hospitalization | Deliveries at age 25 and 32. |
|  | |
| Inclusive sexual and reproductive history | |
| Sexual practices  Sexual partners  Protection: Use of safer sex practices  Use of birth control if appropriate  Risk of intimate partner violence | Any answer acceptable. |
| OB/GYN history | G2P2, normal pregnancies with C-section deliveries at age 25 and 32. At 35 years old you had a tubal ligation. |
| Medications | Tylenol 500mg x 4 pills per day  Thyroid hormones daily |
| Immunizations | - Tetanus - Flu - Hepatitis - Pneumovax - HPV - Other |
| Tobacco products   - Cigarettes - Cigar - Pipe - Chew - E-cigarettes | - Never - Past - year started/year quit - Current   - Quantity   - # of years |
| Alcohol   - Beer - Wine - Liquor - Other | - Never - Past - year started/year quit - Current   - 2 glasses of wine per day   - 30 years |
| Drugs   - Weed - Cocaine - Heroin - Meth - IV - Inhalants - Other | - Never - Past - year started/year quit - Current   - Quantity   - # of years |
| Diet (describe) | Any answer acceptable. |
| Exercise (describe) | Any answer acceptable. |
| List any other important social history or information important to this case | None. |
| Family history |  |
| Mother, father, siblings, grandparents, and other significant findings | Your father died when he was 55 years old due to pancreatic cancer. Your mother is alive and healthy. |
|  |  |
| Physical Exam - List exam maneuvers expected for this case and any abnormal findings that SP will simulate. (tenderness, hyper-hypo reflex, rebound, weakness, etc.)  Fernanda will appear visibly uncomfortable during the encounter. She will occasionally scratch her skin during the visit and hold her right upper belly to express pain.  There is no physical examination during this case. | |
| PHYSICAL EXAM FINDINGS |  |
| 1. Written in layperson’s terms |  |
| 1. General appearance - affect, appearance, position of patient at opening (i.e., sitting, lying down, holding abdomen, etc.) | When the student joins the video call you should be sitting in a chair wearing pajamas and a coat (or your regular clothes). |
| 1. Vital signs | T: 98.2° F  Pulse: 75 bpm  BP: 125/64  RR: 22 |
| 1. Specific findings and affect | Fernanda will appear visibly uncomfortable during the encounter. |
| 1. Response to certain physical movements | Fernanda will occasionally scratch her skin during the visit and hold her right upper belly to express pain. |
|  |  |
| DIAGNOSIS AND DIFFERENTIAL |  |
| Diagnosis with support from positive and negative history and PE findings | Acute viral hepatitis (probably hepatitis A) |
| Differential with support from positive and negative history and PE findings | Gallstones or biliary stenosis, primary biliary cirrhosis, drug-induced hepatitis, undiagnosed chronic hepatitis (such as hepatitis C). |
|  |  |
| MANAGEMENT OR DIAGNOSTIC PLAN | Explain the possible causes, mentioning that a diagnostic evaluation will be conducted, including serological tests for hepatitis A, B, and C, liver function tests, and an abdominal ultrasound to assess the bile ducts and rule out obstruction. If viral hepatitis is confirmed, supportive management will be provided, including hydration and monitoring of liver function. |
|  |  |
| PROFESSIONALISM ISSUES OR CHALLENGES | Cultural competency. |
