# Supplementary material for: A Standardized Clinical Case-Based Assessment for Evaluating Medical Students' Oral Spanish Communication Skills
Source: MedEdPORTAL. 2025 Apr 17;21:11518. doi: 10.15766/mep_2374-8265.11518 (PMC12003672; doi:10.15766/mep_2374-8265.11518)
Supplement: Supplementary file 1 — Precourse Self-Assessment Video.mp4Patient-Provider Interaction Checklist.docxSP Case Spanish.docxSP Case English.docxSP Pilot Case 1 Spanish.docxSP Pilot Case 1 English.docxSP Pilot Case 2 Spanish.docxSP Pilot Case 2 English.docxSP Pilot Case 3 Spanish.docxSP Pilot Case 3 English.docxFacilitators Guide.docx [file mep_2374-8265.11518-s001.zip › I. SP Pilot Case 3 Spanish.docx]

Appendix I: Standardized Patient Case Development Tool Pilot Case 3 Spanish

Instructions: Facilitator and Standardized Patient should use the Standardized Patient script to conduct the student communication skills assessment

Primary Case Author: Cristina Aguayo-Mazzucato, MD PhD

Secondary Case Author: Brandon Martel

Name of Case: Ictericia en recién nacido

Name of Educational and/or Assessment Activity: Caso piloto de español medico

Type and Level of Learner: Estudiante de español médico de nivel intermedio a avanzado

Patient Name: David Mejía (paciente estandarizado es la madre biológica que lo lleva a consulta y responde las preguntas)

Chief Concern: Ojos y piel amarillos

Most Likely Diagnosis and Differential with Rationale from History and/or Physical Exam: El diagnóstico más probable para este paciente es ictericia fisiológica neonatal. Esto se debe a que el bebé de 5 días presenta coloración amarilla en la piel y los ojos, que es un síntoma común en los recién nacidos. La ictericia fisiológica suele aparecer entre el segundo y cuarto día de vida y es más frecuente en bebés amamantados, como es el caso de David. Además, la historia familiar de ictericia neonatal en la hermana mayor refuerza la posibilidad de que se trate de una condición fisiológica temporal. Diagnóstico diferencial incluye Ictericia por incompatibilidad de grupo sanguíneo (incompatibilidad ABO) pero es menos probable debido a la coincidencia entre el grupo sanguíneo del bebé y la madre. Ictericia por infección neonatal (sepsis neonatal) dado que la madre tuvo un cultivo positivo para bacterias y recibió antibióticos antes del parto, sin embargo, la ausencia de fiebre, vómitos u otros signos de infección disminuye la probabilidad de este diagnóstico. La atresia biliar, una condición en la cual los conductos biliares están bloqueados o malformados, también puede causar ictericia. Sin embargo, esta suele aparecer más tarde (después de las primeras dos semanas de vida) y suele ir acompañada de heces pálidas, lo cual no se presenta en este caso.

Ictericia por leche materna en bebés amamantados y puede persistir durante varias semanas. Aunque la ictericia por leche materna aparece típicamente después de la primera semana de vida, podría ser una consideración dado que el bebé está siendo alimentado exclusivamente con leche materna.

Domains: Check all that apply

- Professionalism
- Communication and Interpersonal Skills
- Medical History
- Physical Exam
- Shared Decision-Making
- Patient Education
- Clinical Reasoning
- Documentation
- Handoff
- Presentation
- Other:

Case Objectives: Please list specific objectives for each of the domains you have checked above

1. Demostrar empatía y sensibilidad cultural al abordar las preocupaciones de la madre sobre la salud de su recién nacido, tranquilizándola mientras se mantiene una actitud profesional y respetuosa.
2. Establecer una comunicación clara y de apoyo con la madre, escuchando activamente sus inquietudes.
3. Obtener una historia clínica completa y detallada del recién nacido, enfocándose en el historial de nacimiento, antecedentes familiares de ictericia neonatal, patrones de alimentación y cualquier factor materno relevante como infecciones bacterianas o el uso de antibióticos antes del parto.

Standardized Patient Script:

| SETTING: outpatient, in patient, ED, home, nursing home, rehab, group, etc. | Clínica de urgencias pediátricas (paciente estandarizado es la madre biológica que lo lleva a consulta y responde las preguntas). |
| --- | --- |
| PATIENT PROFILE: Information about the “patient” that helps select an SP and helps the learner get an understanding of them as a person. SP will know more information about the patient than learner will ever ask but allows SP to portray a fully developed patient personality. If none of the items below are particulars for the case, please write “Any answer acceptable.” | |
| Age range | 5 días de nacido. |
| Religious/spiritual background | Cualquier respuesta es aceptable. |
| Sex (e.g. male, female, intersex, transwoman, transman) | Masculino. |
| Sexual orientation (e.g. heterosexual, lesbian, gay, bisexual, pansexual, queer, asexual) | N/A |
| Gender expression (e.g. man, woman, genderqueer) | N/A |
| Race and ethnicity (e.g. to promote educational diversity, we use a diverse pool of SPs.) | Persona hispana/latina. |
| Physical description (e.g. BMI, height range) | Cualquier respuesta es aceptable. |
| Physical limitations | Cualquier respuesta es aceptable. |
| Patient appearance (e.g. disheveled, hospital gown, business casual, casual) | El paciente está envuelto en una manta y sostenido por su madre. |
| Moulage + location (e.g. none, bruises, scars, body piercing, tattoos) | Cualquier respuesta es aceptable. |
| Affect (e.g. pleasant, cooperative) | El paciente está tranquilo y duerme sobre su madre. |
| Family group (e.g. who is family, who they live with) | El paciente vive con su madre biológica, su padre y su hermana mayor (2 años). |
| Education | La madre terminó la escuela secundaria. |
| Level of health literacy | La madre tiene un nivel intermedio de alfabetización en salud. |
| Employment, if any - present and past, noting any current stresses | La madre trabaja a tiempo parcial como cuidadora infantil en una guardería; actualmente está de baja por maternidad. |
| Home/homeless - type of dwelling, number of stories, owned or rented | La familia vive en una casa unifamiliar de su propiedad. |
| Financial situation - any current stresses | Cualquier respuesta es aceptable. |
| Insurance status (e.g. un/under/insured, public/private, HMO/PPO) | Cualquier respuesta es aceptable. |
| Habits (i.e., diet, exercise, caffeine, smoking, alcohol, drugs) | El paciente ha estado amamantando bien durante los últimos 5 días. En el hogar no se permite fumar. |
| Activities (i.e., hobbies, sports, clubs, friends) | N/A |
| Typical day - what is the usual daily routine | Cualquier respuesta es aceptable. |

| CASE INFORMATION | |
| --- | --- |
| Chief Concern: What the patient will say when greeted by the student. The patient’s primary reason for seeking medical care often stated in their own words. | “Su piel está empezando a ponerse amarilla, igual que la de su hermana.” |
| Additional Concerns: Other, if any, concerns the patient has today (i.e., symptoms, requests, expectations, etc.) that will become part of set agenda. | Ninguna. |
| THE PATIENT’S STORY: The SP will be asked to tell their symptom story and the personal and emotional impact for each of their concerns. You will want to write this in the patient’s voice. The symptom story should be able to answer this question: “Tell me more about [chief concern/additional concern], starting at the beginning and bringing me up to now.”  The personal context should be able to answer questions concerning the broader personal/psychosocial context of symptoms, especially the patient’s beliefs/attributions.  The emotional context should be able to ask how are you doing with this, how does this make you feel, how has this affected you emotionally? IMPACT: How has this affected your life? How has this been for your family? | “Traje a David hoy porque ayer sus ojos y piel comenzaron a ponerse amarillos. El color no ha cambiado ni se ha extendido más allá de su cara y manos, pero aún estoy preocupada. A su hermana le pasó lo mismo poco después de que nació, y tuvo que quedarse en el hospital durante varios días. Hasta ahora, David no ha vomitado y su caca es normal: suele hacer caca 2 o 3 veces al día (las heces son de color amarillo) y le cambio el pañal 7 u 8 veces al día. Ha estado tomando el pecho bien y produzco suficiente leche para satisfacer su hambre. Parece estar tranquilo y feliz, pero aún estoy preocupada. ¿Los cambios de color pasarán solos o tendremos que ir al hospital nuevamente, como con su hermana?” |
| HISTORY OF PRESENT ILLNESS: Although some of the HPI will be given in the patient’s symptom story, the learners will expand the story during the direct question section. Below, describe the detailed history, usually about the chief concern, which the student must develop in order to make a useful assessment of the problem: | |
| Onset (when; gradual or sudden) | Hace 1 día (a los 4 días de edad); inicio rápido. |
| Setting (what was going on or where was patient when symptoms first noticed?) | Poco después del nacimiento. |
| Duration (how long) | 1 día hasta ahora. |
| Time relationships (frequency, constant or intermittent) | Constante. |
| Location | Coloración amarillenta de los ojos y de la piel alrededor de la cara y las manos. |
| Radiation | Ninguna. |
| Quality | Coloración amarilla moderada. |
| Amount | N/A |
| Aggravated by what | N/A |
| Relieved by what | N/A |
| Associated with what | N/A |
| Attitude (what does the patient think is the problem, and how do they feel about it) | La madre está preocupada por la decoloración y cree que podría ser necesaria una hospitalización, como en el caso de su hijo anterior. |
| Overall course | Los ojos y áreas de la piel se han vuelto amarillas en el último día. |
| REVIEW OF SYSTEMS: Significant positives and negatives | |
| NEGATIVES | POSITIVES |
| No ha tenido mocos, tos, fiebre, ni respiración anormal. | Coloración amarillenta de los ojos y de la piel alrededor de la cara y las manos. |
| No hay cambios en el apetito, la frecuencia de las deposiciones, la urinación, o la salivación. No hay vómitos y el abdomen no está inflamado. No hay sangre en las heces. | Heces amarillas. |
| No ha tenido convulsiones ni cambios de comportamiento. |  |
|  |  |
| Past medical history |  |
| Medication allergies (name and reaction) | Ninguna. |
| Environmental allergies (name and reaction) | Ninguna. |
| Illnesses | Ninguna. |
| Vaccinations | Lo vacunaron pocas horas después del parto: hepatitis B al nacer y una inyección de Vitamina K. |
| Surgeries | Ninguna. |
| Accidents/injuries/trauma | Ninguna. |
| Hospitalization | Ninguna. |
|  | |
| Inclusive sexual and reproductive history | |
| Sexual practices  Sexual partners  Protection: Use of safer sex practices  Use of birth control if appropriate  Risk of intimate partner violence | N/A |
| OB/GYN history | David nació por parto vaginal sin complicaciones. Se fueron a casa al día siguiente. El embarazo fue a término. La madre tuvo un cultivo positivo para bacterias y le dieron antibióticos antes del parto. |
| Medications | Ninguna. |
| Immunizations | - Tétano - Gripe - Hepatitis - Vacuna antineumocócica - VPH - Otros: COVID |
| Tobacco products   - Cigarrillos - Puros - Pipas - Masticables - Cigarrillos electrónicos | - Nunca - Pasado – año de inicio/año de abandono - Actual   - Cantidad   - # de años |
| Alcohol   - Cerveza - Vino - Licor - Otros | - Nunca - Pasado – año de inicio/año de abandono - Actual   - Cantidad   - # de años |
| Drugs   - Marihuana - Cocaína - Heroína - Metanfetamina - Drogas intravenosas - Inhalantes - Otros | - Nunca - Pasado – año de inicio/año de abandono - Actual   - Cantidad   - # de años |
| Diet (describe) | Leche materna. |
| Exercise (describe) | N/A |
| List any other important social history or information important to this case | Ninguna. |
| Family history |  |
| Mother, father, siblings, grandparents, and other significant findings | Su hermana también se puso amarilla al nacer, la hospitalizaron. El tipo de sangre de David es B+ y sus padres son B+ (madre) y A+ (padre). |
|  |  |
| Physical Exam - List exam maneuvers expected for this case and any abnormal findings that SP will simulate. (tenderness, hyper-hypo reflex, rebound, weakness, etc.)  La madre de David se sentará y lo abrazará durante el encuentro. David estará durmiendo durante la visita.  No se realizará ningún examen físico durante este caso. | |
| PHYSICAL EXAM FINDINGS |  |
| 1. Written in layperson’s terms |  |
| 1. General appearance - affect, appearance, position of patient at opening (i.e., sitting, lying down, holding abdomen, etc.) | Cuando el estudiante se una a la videollamada deberá estar sentado en una silla vistiendo su ropa habitual y sosteniendo a “David” (un muñeco u objeto envuelto en una manta). |
| 1. Vital signs | Temperatura: 97.5° F  Pulso: 135 bpm  Presión arterial: 68/42  Frecuencia respiratoria: 53 |
| 1. Specific findings and affect | David estará durmiendo durante la visita. |
| 1. Response to certain physical movements | N/A |
|  |  |
| DIAGNOSIS AND DIFFERENTIAL |  |
| Diagnosis with support from positive and negative history and PE findings | Ictericia fisiológica neonatal sepsis neonatal |
| Differential with support from positive and negative history and PE findings | Ictericia por incompatibilidad de grupo sanguíneo (incompatibilidad ABO), atresia biliar, ictericia por leche materna |
|  |  |
| MANAGEMENT OR DIAGNOSTIC PLAN | Tranquilizar a la madre y explicar que se realizará un monitoreo cercano de los niveles de bilirrubina para determinar si la ictericia se resuelve por sí sola o si requiere fototerapia. |
|  |  |
| PROFESSIONALISM ISSUES OR CHALLENGES | Competencia cultural. |
