# Supplementary material for: A Standardized Clinical Case-Based Assessment for Evaluating Medical Students' Oral Spanish Communication Skills
Source: MedEdPORTAL. 2025 Apr 17;21:11518. doi: 10.15766/mep_2374-8265.11518 (PMC12003672; doi:10.15766/mep_2374-8265.11518)
Supplement: Supplementary file 1 — Precourse Self-Assessment Video.mp4Patient-Provider Interaction Checklist.docxSP Case Spanish.docxSP Case English.docxSP Pilot Case 1 Spanish.docxSP Pilot Case 1 English.docxSP Pilot Case 2 Spanish.docxSP Pilot Case 2 English.docxSP Pilot Case 3 Spanish.docxSP Pilot Case 3 English.docxFacilitators Guide.docx [file mep_2374-8265.11518-s001.zip › J. SP Pilot Case 3 English.docx]

Appendix J: Standardized Patient Case Development Tool Pilot Case 3 English

Instructions: Facilitator and Standardized Patient should use the Standardized Patient script to conduct the student communication skills assessment

Primary Case Author: Cristina Aguayo-Mazzucato, MD PhD

Secondary Case Author: Brandon Martel

Name of Case: Newborn Jaundice

Name of Educational and/or Assessment Activity: Pilot Case for Medical Spanish

Type and Level of Learner: Intermediate to Advanced Medical Spanish Student

Patient Name: David Mejía (Standardized Patient is biological mother who takes him to the doctor and answers questions)

Chief Concern: Yellow eyes and skin

Most Likely Diagnosis and Differential with Rationale from History and/or Physical Exam: The most likely diagnosis for this patient is neonatal physiological jaundice. This is because the 5-day-old baby presents with yellowing of the skin and eyes, which is a common symptom in newborns. Physiological jaundice typically appears between the second and fourth day of life and is more frequent in breastfed babies, as is the case with David. The family history of neonatal jaundice in the older sister supports the possibility that this is a temporary physiological condition. The differential diagnosis includes jaundice due to blood group incompatibility (ABO incompatibility), but this is less likely due to the matching blood types of the baby and the mother. Jaundice due to neonatal infection (neonatal sepsis) is also a possibility, given that the mother had a positive bacterial culture and received antibiotics before delivery. However, the absence of fever, vomiting, or other signs of infection reduces the likelihood of this diagnosis. Biliary atresia, a condition where the bile ducts are blocked or malformed, can also cause jaundice. However, this typically appears later (after the first two weeks of life) and is usually accompanied by pale stools, which are not present in this case. Breast milk jaundice in breastfed babies can persist for several weeks. Although breast milk jaundice typically appears after the first week of life, it could still be a consideration since the baby is being exclusively breastfed.

Domains: Check all that apply

- Professionalism
- Communication and Interpersonal Skills
- Medical History
- Physical Exam
- Shared Decision-Making
- Patient Education
- Clinical Reasoning
- Documentation
- Handoff
- Presentation
- Other:

Case Objectives: Please list specific objectives for each of the domains you have checked above

1. Demonstrate empathy and cultural sensitivity when addressing the mother's concerns about her newborn's health, reassuring her while maintaining a professional and respectful demeanor.
2. Engage in clear, supportive communication with the mother, actively listening to her concerns and explaining the baby's condition and potential treatments.
3. Obtain a thorough and detailed medical history of the newborn, focusing on birth history, family history of neonatal jaundice, feeding patterns, and any relevant maternal factors such as bacterial infections or antibiotic use before delivery.

Standardized Patient Script:

| SETTING: outpatient, in patient, ED, home, nursing home, rehab, group, etc. | Pediatric urgent care clinic (Standardized Patient is biological mother who takes him to the doctor and answers questions). |
| --- | --- |
| PATIENT PROFILE: Information about the “patient” that helps select an SP and helps the learner get an understanding of them as a person. SP will know more information about the patient than learner will ever ask but allows SP to portray a fully developed patient personality. If none of the items below are particulars for the case, please write “Any answer acceptable.” | |
| Age range | 5 days old. |
| Religious/spiritual background | Any answer acceptable. |
| Sex (e.g. male, female, intersex, transwoman, transman) | Male. |
| Sexual orientation (e.g. heterosexual, lesbian, gay, bisexual, pansexual, queer, asexual) | N/A |
| Gender expression (e.g. man, woman, genderqueer) | N/A |
| Race and ethnicity (e.g. to promote educational diversity, we use a diverse pool of SPs.) | Hispanic/Latinx. |
| Physical description (e.g. BMI, height range) | Any answer acceptable. |
| Physical limitations | Any answer acceptable. |
| Patient appearance (e.g. disheveled, hospital gown, business casual, casual) | Patient is wrapped in blanket and held by mother. |
| Moulage + location (e.g. none, bruises, scars, body piercing, tattoos) | Any answer acceptable. |
| Affect (e.g. pleasant, cooperative) | Patient is calm and sleeps on his mother. |
| Family group (e.g. who is family, who they live with) | Patient lives with biological mother, father, and older sister (2 years old). |
| Education | Mother completed high school. |
| Level of health literacy | Mother has intermediate health literacy. |
| Employment, if any - present and past, noting any current stresses | Mother works as part-time child caregiver at nursery; she is currently on maternity leave. |
| Home/homeless - type of dwelling, number of stories, owned or rented | Family lives in a single unit home that they own. |
| Financial situation - any current stresses | Any answer acceptable. |
| Insurance status (e.g. un/under/insured, public/private, HMO/PPO) | Any answer acceptable. |
| Habits (i.e., diet, exercise, caffeine, smoking, alcohol, drugs) | Patient has breastfed well for the past 5 days. The home is a non-smoking household. |
| Activities (i.e., hobbies, sports, clubs, friends) | N/A |
| Typical day - what is the usual daily routine | Any answer acceptable. |

| CASE INFORMATION | |
| --- | --- |
| Chief Concern: What the patient will say when greeted by the student. The patient’s primary reason for seeking medical care often stated in their own words. | “His skin is starting to turn yellow, just like his sister’s.” |
| Additional Concerns: Other, if any, concerns the patient has today (i.e., symptoms, requests, expectations, etc.) that will become part of set agenda. | None. |
| THE PATIENT’S STORY: The SP will be asked to tell their symptom story and the personal and emotional impact for each of their concerns. You will want to write this in the patient’s voice. The symptom story should be able to answer this question: “Tell me more about [chief concern/additional concern], starting at the beginning and bringing me up to now.”  The personal context should be able to answer questions concerning the broader personal/psychosocial context of symptoms, especially the patient’s beliefs/attributions.  The emotional context should be able to ask how are you doing with this, how does this make you feel, how has this affected you emotionally? IMPACT: How has this affected your life? How has this been for your family? | “I brought David in today because yesterday his eyes and skin started to turn yellow. The color has not changed or spread beyond his face and hands, but I am still worried. The same thing happened to his sister soon after she was born, and she had to stay in the hospital for several days. So far, David has not thrown up and his poop is normal – he usually poops 2-3 times per day (the stool is yellow in color) and I change his diaper 7-8 times per day. He has been breastfeeding well and I make enough milk to match his hunger. He seems to be calm and happy, but I am still worried. Will the color changes pass on their own, or will we have to go to the hospital again, like with his sister?” |
| HISTORY OF PRESENT ILLNESS: Although some of the HPI will be given in the patient’s symptom story, the learners will expand the story during the direct question section. Below, describe the detailed history, usually about the chief concern, which the student must develop in order to make a useful assessment of the problem: | |
| Onset (when; gradual or sudden) | 1 day ago (at 4 days of age); rapid onset. |
| Setting (what was going on or where was patient when symptoms first noticed?) | Shortly after birth. |
| Duration (how long) | 1 day so far. |
| Time relationships (frequency, constant or intermittent) | Constant. |
| Location | Yellowing of eyes and skin around face and hands. |
| Radiation | None. |
| Quality | Moderate yellow discoloration. |
| Amount | N/A |
| Aggravated by what | N/A |
| Relieved by what | N/A |
| Associated with what | N/A |
| Attitude (what does the patient think is the problem, and how do they feel about it) | Mother is worried about the discoloration and believes hospitalization may be required, as with her prior child. |
| Overall course | Eyes and areas of skin have turned yellow in the past day. |
| REVIEW OF SYSTEMS: Significant positives and negatives | |
| NEGATIVES | POSITIVES |
| No runny nose, cough, fever, or abnormal breathing. | Yellowing of eyes and skin around face and hands. |
| No changes to appetite, frequency of bowel movements, urination, or salivation. No vomiting and belly is not swollen. No blood in the stool. | Yellow stool. |
| No seizures and no behavioral changes. |  |
|  |  |
| Past medical history |  |
| Medication allergies (name and reaction) | None. |
| Environmental allergies (name and reaction) | None. |
| Illnesses | None. |
| Vaccinations | David was vaccinated a few hours after birth for hepatitis B and also received a vitamin K injection. |
| Surgeries | None. |
| Accidents/injuries/trauma | None. |
| Hospitalization | None. |
|  | |
| Inclusive sexual and reproductive history | |
| Sexual practices  Sexual partners  Protection: Use of safer sex practices  Use of birth control if appropriate  Risk of intimate partner violence | N/A |
| OB/GYN history | David was a full-term pregnancy and was born via vaginal delivery with no complications; he and his mother both went home the next day. His mother had positive bacterial cultures and was given antibiotics before delivery. |
| Medications | None. |
| Immunizations | - Tetanus - Flu - Hepatitis - Pneumovax - HPV - Other |
| Tobacco products   - Cigarettes - Cigar - Pipe - Chew - E-cigarettes | - Never - Past - year started/year quit - Current   - Quantity   - # of years |
| Alcohol   - Beer - Wine - Liquor - Other | - Never - Past - year started/year quit - Current   - 2 bottles on weekends   - 35 years |
| Drugs   - Weed - Cocaine - Heroin - Meth - IV - Inhalants - Other | - Never - Past - year started/year quit - Current   - Quantity   - # of years |
| Diet (describe) | Breastmilk. |
| Exercise (describe) | N/A |
| List any other important social history or information important to this case | None. |
| Family history |  |
| Mother, father, siblings, grandparents, and other significant findings | David’s sister also developed yellow coloring of the skin after birth that required hospitalization. David’s blood type is B+ and his parents are B+ (mother) and A+ (father). |
|  |  |
| Physical Exam - List exam maneuvers expected for this case and any abnormal findings that SP will simulate. (tenderness, hyper-hypo reflex, rebound, weakness, etc.)  David’s mother will sit holding him during the encounter. David will be sleeping during the visit.  There is no physical examination during this case. | |
| PHYSICAL EXAM FINDINGS |  |
| 1. Written in layperson’s terms |  |
| 1. General appearance - affect, appearance, position of patient at opening (i.e., sitting, lying down, holding abdomen, etc.) | When the student joins the video call you should be sitting in a chair wearing your regular clothes and holding “David” (a doll or object wrapped in a blanket). |
| 1. Vital signs | T: 97.5° F  Pulse: 135 bpm  BP: 68/42  RR: 53 |
| 1. Specific findings and affect | David will be sleeping during the visit. |
| 1. Response to certain physical movements | N/A |
|  |  |
| DIAGNOSIS AND DIFFERENTIAL |  |
| Diagnosis with support from positive and negative history and PE findings | Neonatal physiologic jaundice |
| Differential with support from positive and negative history and PE findings | Jaundice due to blood group incompatibility (ABO incompatibility), biliary atresia, breast milk jaundice. |
|  |  |
| MANAGEMENT OR DIAGNOSTIC PLAN | Reassure the mother and explain that close monitoring of bilirubin levels will be done to determine if the jaundice resolves on its own or if phototherapy is required. |
|  |  |
| PROFESSIONALISM ISSUES OR CHALLENGES | Cultural competency. |
