# Supplementary material for: A Standardized Clinical Case-Based Assessment for Evaluating Medical Students' Oral Spanish Communication Skills
Source: MedEdPORTAL. 2025 Apr 17;21:11518. doi: 10.15766/mep_2374-8265.11518 (PMC12003672; doi:10.15766/mep_2374-8265.11518)
Supplement: Supplementary file 1 — Precourse Self-Assessment Video.mp4Patient-Provider Interaction Checklist.docxSP Case Spanish.docxSP Case English.docxSP Pilot Case 1 Spanish.docxSP Pilot Case 1 English.docxSP Pilot Case 2 Spanish.docxSP Pilot Case 2 English.docxSP Pilot Case 3 Spanish.docxSP Pilot Case 3 English.docxFacilitators Guide.docx [file mep_2374-8265.11518-s001.zip › K. Facilitators Guide.docx]

# Appendix K: Facilitator’s Guide

# Educational Objectives

# By the end of this activity, facilitators will be able to:

# Use a standardized clinical case in Spanish with training materials for a standardized patient.

# Evaluate students’ oral communication skills with a standardized patient in Spanish.

# FACILITATOR INSTRUCTIONS

# Dear Facilitator,

# Thank you for participating as a facilitator for the Medical Spanish end-of-course evaluation. The case is a Spanish speaking patient with chest pain symptoms that is having an urgent care visit. Each student’s session timing and flow are described below. You will be seated outside the room (in-person format) or moderating break-out rooms over Zoom (virtual format). The assigned student can read the “Instructions to Students” either outside the exam room or over screen share.

# Starting the session

# In-person format

# Arrive in advance to set up the evaluation rooms (posting “Instructions to Students” on the room doors).

# If standardized patients (SPs) require orientation, then the facilitator and SPs should consider arriving at the evaluation rooms several minutes early for orientation.

# Virtual format

# Have important contacts by you in case of technical issues.

# Have your group meeting link available. There will be a single link for all students to log onto. Later, they will be sorted into individual breakout rooms with their SP and facilitator.

# Check that the meeting link is set up with you (facilitator) already as the host. If someone else created the meeting, then they will have to join the meeting and transfer the host role to you. To transfer hosts: The initial host should click on "participants" at the bottom and hover over the name of the person to be made host and then select from the drop-down.

# If SPs require orientation, then the facilitator and SPs should consider arriving at the meeting several minutes early for orientation.

# Orienting the SPs

# If SPs require orientation, then the facilitator should briefly spend several minutes to:

# In-person format

# Introduce yourself to the SPs.

# Make sure the lighting, positioning of the SPs, and evaluation room layout are optimal.

# Let them know you need some minutes to orient the students and assign an interview order.

# Leave the SPs in their evaluation rooms.

# Virtual format

# Introduce yourself to the SPs. You may need to change SP’s name label on Zoom to the case name (e.g. Camilo/a Pérez). You can do this as the host by hovering over the participant and dropping down to rename.

# Make sure the lighting, positioning of the SPs, and audio are optimal.

# Let them know you need some minutes to orient the students and assign breakout rooms.

# Then put SPs back in the virtual waiting room by hovering and selecting “put back in the waiting room”.

# Orienting the students

# In-person format

# Allow students to enter the space outside the evaluation rooms.

# Introduce yourself to the students as the facilitator and explain the station procedures time flow (see “Conducting and Debriefing the SP Encounter” below). At this point, you may select the interview order of students.

# Review the “Instructions to Students” note with students on the outside of the evaluation room door.

# Virtual format

# Allow students to enter from the meeting waiting room into the virtual room.

# Introduce yourself to the students as the facilitator and explain the station procedures time flow (see “Conducting and Debriefing the SP Encounter” below). At this point, you may select the interview order of students.

# Review the “Instructions to Students” note with students over screen share.

# Conducting and debriefing the standardized patient encounter

# Have the selected students enter the evaluation room (in-person format) or assigned breakout room (virtual format) while the other students remain outside the room (in-person) or in the general virtual room (virtual).

# Observation | TIME: MAXIMUM 15 MINUTES

# Observe the student’s discussion with the Standardized Patient and complete the checklist (Appendix C). Please note the station schedule and make sure to end this section on time.

# Feedback | TIME: MAXIMUM 5 MINUTES

# Conduct a feedback discussion with the student, offering the student feedback on key points in any of these domains:

# Ability to build rapport/communication.

# History taking: ability to obtain and understand information.

# Cultural knowledge/sensitivity: understanding that a patient’s culture and beliefs influence interactions and disease. Examples of questions students might ask that demonstrate this knowledge: "How has moving from Venezuela affected your social and work life?", "Can you describe any challenges you face at work with your new supervisor?", and "Do cultural differences affect how you get along with people at work and at home?".

# Ability to build an alliance with the patient to support the patient’s wishes while providing safe and effective care.

# *Example Facilitator Feedback*: “Congratulations on your excellent performance during the medical interview. You covered all/most of the assessment points. You addressed key concerns such as pain characteristics, and family history; the interview flowed nicely, etc.”

# *Example SP Feedback*: "As a patient, I felt listened to and cared for, you showed empathy and respect..."

# Request the student feedback using the following questions:

# What did you learn from this patient?

# Any belief or concept that caught your attention?

# Release the student. Before moving on to the next student, ensure all items on the checklist (Appendix C) are completed (make sure every checklist item is filled with “YES” or “NO”).

# Ending the session

# In-person format

# Instruct released students that they may leave after they receive feedback.

# Complete SP cases with all remaining students, inviting them into the evaluation room.

# Once all students have complete the SP case, you can release the SPs and close up the evaluation space, removing the “Instructions to Students” note.

# Virtual format

# Instruct released students that they may leave after they receive feedback.

# Complete SP cases with all remaining students, inviting them into the appropriate breakout room.

# Once all students have complete the SP case, you can release the SPs and close the Zoom meeting.

# STATION: INSTRUCTIONS FOR THE STUDENT

# Your patient: Camilo/a Perez, a 54-year-old, who arrives to Urgent Care at 2pm due to chest pain. Their preferred language is Spanish.

# You are the student on the admitting team and have been paged to see the patient. You will now be meeting them for the first time.

# Obtain a directed clinical history in Spanish.
