# Supplementary material for: A Standardized Clinical Case-Based Assessment for Evaluating Medical Students' Oral Spanish Communication Skills
Source: MedEdPORTAL. 2025 Apr 17;21:11518. doi: 10.15766/mep_2374-8265.11518 (PMC12003672; doi:10.15766/mep_2374-8265.11518)
Supplement: Supplementary file 1 — Precourse Self-Assessment Video.mp4Patient-Provider Interaction Checklist.docxSP Case Spanish.docxSP Case English.docxSP Pilot Case 1 Spanish.docxSP Pilot Case 1 English.docxSP Pilot Case 2 Spanish.docxSP Pilot Case 2 English.docxSP Pilot Case 3 Spanish.docxSP Pilot Case 3 English.docxFacilitators Guide.docx [file mep_2374-8265.11518-s001.zip › _Educational Summary Report_11518.pdf]

MedEdPORTAL® The AAMC Journal of Teaching and Learning Resources

Original Publication Open Access

A Standardized Clinical Case-Based Assessment for Evaluating Medical Students' Oral Spanish Communication Skills

Margot Mai, AM, Rose L. Molina, MD, MPH, Cristina Aguayo-Mazzucato, MD, PhD, Alejandro A. Diaz, MD, Katia Canenguez, PhD, EdM, Hong Chen Cheung, MD, MPH, Nina Rivera Graupera, MD, Brandon Martel, Silvana Bonilla, MD, MS*

*Corresponding author: silvana.bonilla@childrens.harvard.edu

Abstract

Introduction: Few standardized assessments exist for evaluating medical students' oral Spanish communication with patients. Educators in Medical Spanish need practical, accessible tools. We designed and implemented a standardized clinical case-based assessment to evaluate students' oral Spanish communication skills upon course conclusion. **Methods:** The students enrolled in the Intermediate and Advanced Medical Spanish courses during the Fall 2021 term were included. Students participated in a standardized clinical case-based assessment with a standardized patient. The students' performance was evaluated using scoring of a binary checklist focused on 20 key components of the medical interview in Spanish, and 2 formative feedback items about their Spanish language skills. **Results:** Twenty-eight students participated in the educational activity, including 13 from the Intermediate and 15 from the Advanced Medical Spanish courses. Of the participants, 43% ($n = 12$) were female, 89% ($n = 25$) were medical students and 11% ($n = 3$) were dental students, and 78% ($n = 22$) were first-year students at Harvard Medical School. Overall, students in both the Intermediate and Advanced levels achieved high aggregate performance scores, at or above 80%. Mean scores differed between the two groups ($p = .0001$). **Discussion:** The standardized clinical case-based assessment effectively evaluated students' communication skills upon completing the longitudinal Medical Spanish course. It helped students ask key medical history questions in Spanish. Our curriculum offers a standardized patient case as a model for other instructors to assess oral medical Spanish communication skills.

Keywords
Medical Spanish, Cased-Based Learning, Clinical Skills Assessment/OSCEs, Communication Skills, Standardized Patient, Language-Appropriate Health Care

Educational Objectives

By the end of this activity, learners will be able to:

1. Conduct a medical interview in Spanish with a standardized patient, addressing key components of the clinical encounter.
2. Receive formative feedback provided by facilitators and standardized patients to improve their Medical Spanish communication skills.

Citation:
Mai M, Molina RL, Aguayo-Mazzucato C, et al. A standardized clinical case-based assessment for evaluating medical students' oral Spanish communication skills. *MedEdPORTAL.* 2025;21:11518.
https://doi.org/10.15766/mep_2374-8265.11518

Introduction

Language diversity in the United States continues to change rapidly. The number of households speaking a language other than English at home has increased more than two-fold since the 1990s.[1] In 2022, an estimated 21.7% of people reported preferring a language other than English,[2] with Spanish remaining the most commonly spoken non-English language in the US.[1-4] Denoting non-English language preference focuses on the strengths that patients bring to their health care and reinforces the importance of languages other than English. The growing reality of language diversity highlights the need for health care professionals to be appropriately trained and prepared to address the linguistic and cultural needs of patients who prefer languages other than English.[5,6]

Efforts to improve clinician–patient communication have been actively supported by the National Institute on Minority Health and Health Disparities (NIMHD).[6] The quality of clinician–

Copyright © 2025 Mai et al. This is an open-access publication distributed under the terms of the Creative Commons Attribution license.

patient communication is associated with improved health outcomes, medical adherence, and patient satisfaction.[1, 6-9] Language-concordant care, defined as direct communication between patients and providers in the same language, has also been shown to impact the quality of care and patient outcomes.[10-14] These findings are of particular relevance for Spanish-speaking patients in the US who face many health disparities (e.g., increased rates of cardiovascular disease, Type 2 diabetes, obesity, HIV, and liver disease) and have overall higher mortality rates than the national average.[3,15] In light of these outcomes, many medical schools across the US offer medical language courses, with an estimated two-thirds offering a Medical Spanish curriculum.[16-18] In addition to improvements in listening comprehension, oral skills, fluency, and comfort working with Spanish-speaking patients,[19,20] completion of a medical language course may prepare students to practice language-concordant care in their careers.[21]

Medical education is an optimal setting to study and refine student communication skills in languages other than English. Fostering communication skills improves medical student performance in several domains, including building a relationship with patients and performing clinical assessments.[22] A recent review article spanning the past 20 years highlights the growing body of literature on Medical Spanish educational innovations and assessment tools.[23] Validated instruments such as the Comunicación y Habilidades Interpersonales (CAI) scale[24] and the Physician Oral Language Observation Matrix (POLOM)[25] represent significant advancements in the assessment of Medical Spanish proficiency and cultural sensitivity.[23] Romero Arocha et al. also highlight significant gaps in evaluation data regarding course effectiveness and in the use of pedagogical frameworks to guide curricula.[23]

Since the early 2000s, the Medical Language Program at Harvard Medical School (HMS) has offered Intermediate- and Advanced-level, semester-long courses in Medical Spanish to medical and dental students. HMS alumni reported improved language proficiency following the course and suggested that a formal, end-of-course assessment could benefit student learning.[21] We designed and implemented an end-of-course assessment using a standardized clinical case and a checklist focused on assessing the completion of key components of the medical interview in Spanish during standardized patient encounters. The assessment materials were refined through repeated iterations of our course to ensure their practicality and relevance. Our primary objective is to increase access to Spanish language resources by providing readily implementable tools that faculty at other institutions can use to integrate Medical Spanish assessments into their curricula.

Methods

Medical Spanish Courses

The Medical Spanish courses at HMS focus on three key elements: (1) Latino/Hispanic cultural humility and sensitivity; (2) medical vocabulary for clinical history taking and physical examinations; and (3) communication skills. The Advanced and Intermediate course curricula comprise 13 weekly, 90-minute didactic sessions, focusing on practical and conversational skills. The courses are graded pass/fail, with attendance and active participation being mandatory. The topics for the sessions include different components of the medical interview, including greeting and addressing the patient; building rapport; chief concern; history of present illness; past medical history and surgical history; review of systems, medications, and allergies; family history; social history; and preventive health and mental health. A session on learning to collaborate with medical interpreters is also included. Generally, the sessions start with an ice breaker, followed by a concise review of terminology to be used during the session, with the remainder of the session spent in small-group breakout rooms where the students practice clinical cases that emphasize the topic covered during the session with an instructor. The recommended course textbook is *Spanish and The Medical Interview: A Textbook for Clinically Relevant Medical Spanish* by Dr. Pilar Ortega.[27] The curriculum is organized to ensure that students are exposed to the corresponding components of the medical interview in English before learning it in Spanish.

Students completed a precourse self-assessment to determine their readiness, which involved watching a short video in Spanish (Appendix A) and answering questions about their comprehension and comfort level (e.g., “What percentage of the video did you understand?”; “How comfortable are you taking a class conducted entirely in Spanish?”; and “Have you taken Spanish classes before?”). Students who indicated that they were not comfortable taking a course conducted entirely in Spanish were not admitted, as full immersion was a key component. However, prior experience with Spanish classes was not a requirement for admission. Placement was determined based on the percentage of the video understood. Students who reported understanding at least 70% of the video were assigned to the Advanced course, while those who understood <70% were placed in the Intermediate course. This cutoff was established based on prior course experience and was found to be effective for placing students at the level that best supported their learning.

Copyright © 2025 Mai et al. This is an open-access publication distributed under the terms of the Creative Commons Attribution license.

Facilitators

The facilitators were the Medical Spanish course instructors—all Spanish-speaking physicians with native-level proficiency and experience in teaching Medical Spanish and guiding learners in clinical communication. Their primary responsibilities included observing the student during the encounter with the standardized patient (SP), scoring the Patient-Provider Interaction Checklist (Appendix B) to assess whether the student had completed key components of the medical interview in Spanish, and providing detailed feedback on the student's performance. During the Fall 2021 session, the Advanced course had one instructor and four native-level proficiency Spanish-speaking physician teaching assistants. Each of the three Intermediate courses was led by one instructor with support from three native-level proficiency Spanish-speaking teaching assistants.

Standardized Patients

The SPs were volunteers recruited by the instructors from their respective hospitals. The recruitment process was mostly done through hospital-wide and university-wide listserv emails. Their primary responsibilities included reading the standardized clinical case and patient script to simulate a real patient encounter, and providing feedback about their experience, focusing on how they felt as patients and on the student's communication skills. Most SPs were already involved in the course as teaching assistants, and the main recruitment criterion was native-level proficiency. The seven SPs were native-level Spanish-speaking individuals: five physicians and two medical students. SPs practiced mock interviews with the instructors, responding to potential questions as suggested in the script. SPs were allowed to refer to the written material during the assessment session.

Standardized Clinical Case

We developed a standardized clinical case outlined by a lead instructor and then circulated it amongst the rest of the Medical Spanish course instructors. Each instructor reviewed the content and wording of the case, and a final version was agreed upon by consensus. The clinical case included the following elements: chief concern; medical and surgical history; medications; allergies; family and social history; and health beliefs. An SP script was created based on the case in Spanish and English (Appendices C and D).

End-of-Course Assessment

We designed the end-of-course assessment alongside a checklist comprising 20 items corresponding to the different components of the medical interview. Each item on the checklist was awarded 0 points if the student failed to perform the required action, and 1 point if the student made a clear and effective effort to perform the required action (Appendix B). To ensure that students had a clear understanding of the criteria on which they would be evaluated, the checklist was shared at the beginning of the course when the topics to be covered and the assessment process were outlined. The checklist was completed by the facilitator. This scoring instrument was tested through a pilot program conducted during the Fall 2019 term. The 2019 pilot assessment consisted of six stations with diverse clinical scenarios and was presented as an in-person, end-of-course assessment where students rotated through each of the clinical stations, were assessed on the completion of the different sections of the medical interview, and received an aggregate performance score at the end of the assessment. We include the 3 pilot assessment cases separately (Appendices E-J).

For logistical coordination, we administered the standardized clinical case-based assessment virtually, conducted on the Zoom platform due to the COVID-19 pandemic. All students received written and verbal instructions within 1 week before and on the assessment day. We divided students into rotating groups for the timely completion of the assessment. For example, in the Advanced course, the 15 students were divided into three groups of five students each. Each of the students in a group was placed in one of five breakout rooms along with an SP and a facilitator. All participants had their cameras on. The allotted time was 20 minutes, with 15 minutes for the interview and 5 minutes for feedback. A designated facilitator sent a wrap-up notification to all breakout rooms 1 minute before the time allotted before the interview ended. Feedback was then given to each student by the facilitator and SP. The feedback covered clinical, cultural, and communication aspects and was structured using the standardized checklist. Once the session finished for the first five-student group, the second and third groups followed, completing sessions for all 15 students in approximately 1 hour.

To help future facilitators implement this activity, Appendix K lists the educational objectives, scripts, guidelines, and specific instructions to be used as guidance.

This activity was reviewed and approved by the HMS Program in Medical Education's Educational Scholarship Review Committee and was determined not to meet the definition of research, thereby exempting it from formal institutional review board review.

Results

In the Fall 2021 term, 28 students enrolled in Intermediate ($n = 13$) or Advanced ($n = 15$) Medical Spanish courses and

Copyright © 2025 Mai et al. This is an open-access publication distributed under the terms of the Creative Commons Attribution license.

participated in the standardized clinical case-based assessment, of which 89% ($n = 25$) were medical students and 11% ($n = 3$) were dental students, and 43% ($n = 12$) were female. The majority ($n = 22$, 78%) were first-year students, including all those in the Advanced course. Of the remaining six students, four were fourth-year medical students, and two were first-year dental students.

The overall aggregate performance scores were high for the Advanced- and Intermediate-level student groups, scoring at or above 80%. Mean performance scores differed between the two groups ($p = .0001$; Figure 1). There was no significant difference in scores based on the year of medical school, suggesting that cumulative medical knowledge and experience did not influence the results.

Figure 2 illustrates the percentage of correct completion of each item on the checklist for all students, with item 4 (*Check the patient's identity, e.g., date of birth, age*), item 5 (*Start with small talk*), item 10 (*Ask about the impact that the current disease has on the life of the patient*), and item 11 (*Reviews steps of the visit with the patient*) showing a higher percentage of incorrect responses.

When comparing the 2021 virtual assessment with the 2019 in-person assessment, we found no statistically significant difference in mean performance scores between the virtual and in-person testing formats (Figure 3). Our virtual versus in-person format comparison was based on two groups of three medical students ($N = 6$) who had participated in both the 2019 pilot program and the 2021 end-of-course assessment as students of the Intermediate Medical Spanish course.

Discussion

Our educational activity provides practical assessment materials that faculty can utilize to assess students' interactions with Spanish-speaking patients in SP encounters or as part of Medical Spanish language courses. Currently, existing materials in Spanish are scattered, are not widely available, often require payment, and lack standardization or structured testing.[17,18,26] Our activity offers an openly accessible set of pilot-tested

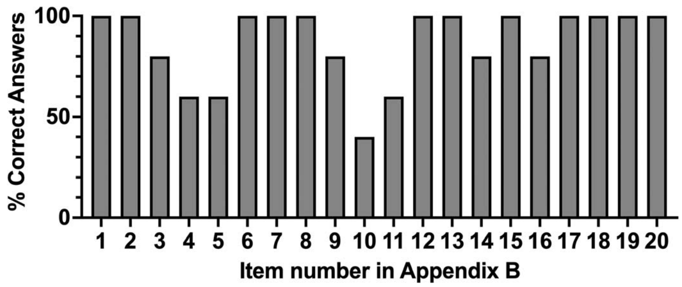

Figure 2. Percentage of correct completion of each item on the Patient-Provider Interaction checklist for all students (Appendix B). Checklist items: (1) Initial greeting; (2) Provider self-introduction; (3) Confirm patient name; (4) Check patient identity; (5) Small talk; (6) Ask open-ended questions; (7) Chief concern; (8) Current illness; (9) Illness beliefs; (10) Illness life impact; (11) Review visit steps; (12) Medical/surgical history; (13) Medications/allergies/adverse reactions; (14) Complementary medicines; (15) Family history; (16) Signs/symptoms; (17) Health behaviors; (18) Social factors; (19) Rapport/empathy; (20) Respectful ending.

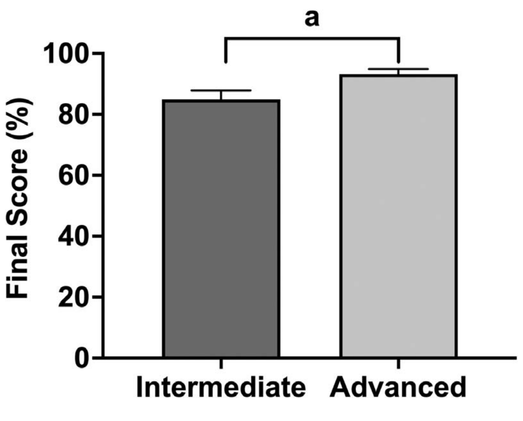

Figure 1. Effects of course level on overall aggregate performance scores. Final assessment scores (expressed as percentages) were assigned by facilitators to students in the Advanced and Intermediate Medical Spanish courses ($n = 75$ scores obtained from students in the Advanced course; $n = 65$ scores obtained from students in the Intermediate course). Bars and whiskers show the *M* and *SD*. [a]$p < .001$, unpaired *t* test.

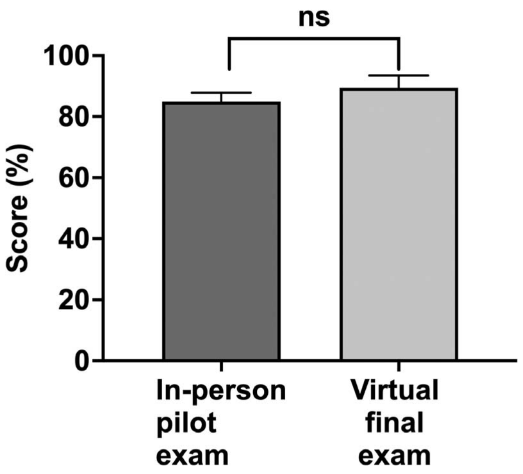

Figure 3. Effects of test format on overall aggregate performance scores. Final assessment scores (expressed as percentages) were assigned by facilitators to students during the 2019 in-person pilot assessment ($N = 6$) and during the 2021 virtual end-of-course assessment ($N = 28$). Bars and whiskers show the *M* and *SD*. Abbreviation: ns, difference not significant.

Copyright © 2025 Mai et al. This is an open-access publication distributed under the terms of the Creative Commons Attribution license.

resources designed to serve as a tool for assessing key components of the medical interview in Spanish.

Educators in the growing academic field of Medical Spanish need practical and accessible educational tools. Ensuring that Medical Spanish courses effectively meet their educational goals and help learners achieve the desired proficiency is a key priority in medical education.[23] Demonstrating their effectiveness is essential to validating their impact and securing ongoing support and development. Our checklist is designed as an accessible resource for assessing specific components of the medical interview in Spanish within the context of SP encounters. Its primary strength lies in its usability and adaptability for educators seeking to integrate Spanish communication assessments into their curricula without the resource-intensive requirements of other tools.

The high completion of most checklist items supports the conclusion that the longitudinal course effectively enables students to ask key medical history questions in Spanish. Students in the Advanced Medical Spanish group scored higher than the Intermediate-level students, likely reflecting the superior language and communication skills of students in the Advanced-level group, attributable to their greater fluency and ability to navigate complex medical conversations in Spanish. However, we found no significant differences in performance scores based on the medical school year, suggesting that more medical knowledge was not a determinant of performance and that the assessment primarily targeted language and communication skills. Alternatively, the high overall scores and lack of distinction between learners in earlier and later years of training may indicate that the binary checklist, while effective in assessing task completion, is not sufficiently nuanced to differentiate varying levels of language proficiency.

A common theme among the checklist items performed incorrectly is their focus on effective communication and patient interaction. Verifying the patient's identity, initiating small talk to build rapport, understanding the impact of the disease on the patient's life, and reviewing the steps of the visit to ensure clarity and set expectations were areas where respondents struggled more. These challenges may reflect common difficulties faced by first-year medical students in patient encounters. Verifying a patient's identity, for instance, may be overlooked by students, due to their limited clinical exposure and focus on content rather than procedural details. Small talk, on the other hand, can be particularly challenging in a second language, as it requires spontaneous, culturally nuanced conversation that is often less rehearsed and overlooked in early clinical training. Addressing the impact of the disease on the patient's life and reviewing the steps of the visit demand higher-order skills, such as patient-centered communication and agenda setting, which are underdeveloped in early learners and further complicated by the cognitive load of bilingual communication. These findings suggest the need for enhanced training or awareness of these components of patient care.

Limitations of our educational activity include the selection of SPs. Because funding for hiring professional SPs was lacking, we recruited volunteer SPs without compensation or comprehensive training. Most of these SPs were either physicians or medical students already serving as teaching assistants for the courses, which is not representative of the broader patient demographic that students are likely to encounter in their medical careers. For future implementations, we recommend recruiting SPs with more diverse backgrounds and, when possible, compensating professional SPs to enhance the realism and representativeness of the encounters. Another limitation is the use of a binary checklist, which, while effective for assessing task completion, lacks the nuance needed to differentiate varying levels of language proficiency. Additionally, no reliability data were available, as only one facilitator completed the checklist. Future work should include a larger and more varied sample of learners, along with the development of a more detailed scoring rubric that assesses performance on a continuum. This approach would provide a more comprehensive assessment of Spanish proficiency and communication skills and better differentiate between learner levels.

We view the virtual format of the assessment and curriculum not as a limitation but as a factor that enhances accessibility. Since the COVID-19 pandemic, our program has delivered Medical Spanish courses virtually, requiring fundamental changes to how we approach education. We believe that the virtual format has increased accessibility, allowing more students to enroll in and benefit from the course. This semester, we are transitioning to a hybrid format, and we continue to observe strong interest and enrollment.

In conclusion, our clinical case-based assessment provides a needed, free-of-charge set of pilot-tested materials designed to serve as a tool for assessing key components of the medical interview in a Medical Spanish program. By sharing our experience, we hope to provide a resource for other Medical Spanish instructors to use and modify as needed to fit their curricula.

Copyright © 2025 Mai et al. This is an open-access publication distributed under the terms of the Creative Commons Attribution license.

Appendices

A. Precourse Self-Assessment Video.mp4

B. Patient-Provider Interaction Checklist.docx

C. SP Case Spanish.docx

D. SP Case English.docx

E. SP Pilot Case 1 Spanish.docx

F. SP Pilot Case 1 English.docx

G. SP Pilot Case 2 Spanish.docx

H. SP Pilot Case 2 English.docx

I. SP Pilot Case 3 Spanish.docx

J. SP Pilot Case 3 English.docx

K. Facilitators Guide.docx

All appendices are peer reviewed as integral parts of the Original Publication.

Margot Mai, AM: Third-Year Medical Student, Harvard Medical School; Fourth-Year Doctoral Student, Department of Anthropology, Harvard Graduate School of Arts and Sciences

Rose L. Molina, MD, MPH: Assistant Professor of Obstetrics, Gynecology & Reproductive Biology, Harvard Medical School; Physician, Department of Obstetrics and Gynecology, Beth Israel Deaconess Medical Center

Cristina Aguayo-Mazzucato, MD, PhD: Assistant Professor of Medicine, Harvard Medical School; Assistant Investigator, Section on Islet Cell & Regenerative Biology, Joslin Diabetes Center

Alejandro A. Diaz, MD: Assistant Professor of Medicine, Harvard Medical School; Physician, Division of Pulmonary and Critical Care Medicine, Brigham and Women's Hospital

Katia Canenguez, PhD, EdM: Instructor in Psychiatry, Harvard Medical School; Pediatric Psychologist, Department of Psychiatry, Massachusetts General Hospital

Hong Chen Cheung, MD, MPH: Instructor in Medicine, Brigham and Women's Hospital

Nina Rivera Graupera, MD: Assistant Professor of Psychiatry, Harvard Medical School; Attending Psychiatrist, Department of Psychiatry and Behavioral Sciences, Boston Children's Hospital

Brandon Martel: Summer Intern, Section on Islet Cell & Regenerative Biology, Joslin Diabetes Center

Silvana Bonilla, MD, MS: Assistant Professor of Pediatrics, Division of Pediatric Gastroenterology, Hepatology and Nutrition, Boston Children's Hospital; ORCID: https://orcid.org/0000-0001-6665-825X

Acknowledgments
The authors would like to thank Kari Hannibal for her support during the course and her useful suggestions for this curriculum.

Disclosures
None to report.

Funding/Support
None to report.

Ethical Approval
The Harvard Medical School Program in Medical Education's Educational Scholarship Review Committee reviewed this project.

References

1. McCardle P, Leung CYY. English language learners: development and intervention: an introduction. *Top Lang Disorders*. 2006;26(4):302-304. https://doi.org/10.1097/00011363-200610000-00003
2. DP02: selected social characteristics in the United States. US Census Bureau. Accessed March 11, 2025. https://data.census.gov/table/ACSDP5Y2022.DP02?=&hidePreview=true
3. Vega WA, Rodriguez MA, Gruskin E. Health disparities in the Latino population. *Epidemiol Rev*. 2009;31(1):99-112. https://doi.org/10.1093/epirev/mxp008
4. Batalova J, Zong J. Language diversity and English proficiency in the United States. Migration Policy Institute. November 11, 2016. Accessed March 11, 2025. https://www.migrationpolicy.org/article/language-diversity-and-english-proficiency-united-states
5. Callahan E, Garcia E, Rehm J. Talk louder? Communicating with your Spanish speaking patients. *MedEdPORTAL*. 2011;7:8427. https://doi.org/10.15766/mep_2374-8265.8427
6. Steinberg EM, Valenzuela-Araujo D, Zickafoose JS, Kieffer E, DeCamp LR. The "battle" of managing language barriers in health care. *Clin Pediatr (Phila).* 2016;55(14):1318-1327. https://doi.org/10.1177/0009922816629760
7. Ortega P. Spanish language concordance in U.S. medical care: a multifaceted challenge and call to action. *Acad Med*. 2018;93(9): 1276-1280. https://doi.org/10.1097/ACM.0000000000002307
8. Fernández A, Pérez-Stable EJ. ¿Doctor, habla español? Increasing the supply and quality of language-concordant physicians for Spanish-speaking patients. *J Gen Intern Med*. 2015;30(10): 1394-1396. https://doi.org/10.1007/s11606-015-3436-x
9. Haskard Zolnierek KB, Dimatteo MR. Physician communication and patient adherence to treatment: a meta-analysis. *Med Care*. 2009;47(8):826-834. https://doi.org/10.1097/MLR.0b013e31819a5acc
10. Stewart MA. Effective physician–patient communication and health outcomes: a review. *CMAJ*. 1995;152(9):1423-1433.
11. Ha JF, Longnecker N. Doctor-patient communication: a review. *Ochsner J*. 2010;10(1):38-43.
12. Stewart M, Brown JB, Boon H, Galajda J, Meredith L, Sangster M. Evidence on patient-doctor communication. *Cancer Prev Control*. 1999;3(1):25-30.

Copyright © 2025 Mai et al. This is an open-access publication distributed under the terms of the Creative Commons Attribution license.

13. Diamond L, Izquierdo K, Canfield D, Matsoukas K, Gany F. A systematic review of the impact of patient-physician non-English language concordance on quality of care and outcomes. *J Gen Intern Med*. 2019;34(8):1591-1606. https://doi.org/10.1007/s11606-019-04847-5
14. Lor M, Martinez GA. Scoping review: definitions and outcomes of patient-provider language concordance in healthcare. *Patient Educ Couns*. 2020;103(10):1883-1901. https://doi.org/10.1016/j.pec.2020.05.025
15. Aguayo-Mazzucato C, Diaque P, Hernandez S, Rosas S, Kostic A, Caballero AE. Understanding the growing epidemic of type 2 diabetes in the Hispanic population living in the United States. *Diabetes Metab Res Rev*. 2019;35(2):e3097. https://doi.org/10.1002/dmrr.3097
16. Molina RL, Kasper J. The power of language-concordant care: a call to action for medical schools. *BMC Med Educ*. 2019;19(1): 378. https://doi.org/10.1186/s12909-019-1807-4
17. Morales R, Rodriguez L, Singh A, et al. National survey of medical Spanish curriculum in U.S. medical schools. *J Gen Intern Med*. 2015;30(10):1434-1439. https://doi.org/10.1007/s11606-015-3309-3
18. Ortega P, Francone NO, Santos MP, et al. Medical Spanish in US medical schools: a national survey to examine existing programs. *J Gen Intern Med*. 2021;36(9):2724-2730. https://doi.org/10.1007/s11606-021-06735-3
19. Ortega P, Park YS, Girotti JA. Evaluation of a medical Spanish elective for senior medical students: improving outcomes through OSCE assessments. *Med Sci Educ*. 2017;27(2):329-337. https://doi.org/10.1007/s40670-017-0405-5
20. Reuland DS, Frasier PY, Slatt LM, Aleman MA. A longitudinal medical Spanish program at one US medical school. *J Gen Intern Med*. 2008;23(7):1033-1037. https://doi.org/10.1007/s11606-008-0598-9
21. Pereira JA, Hannibal K, Stecker J, Kasper J, Katz JN, Molina RL. Professional language use by alumni of the Harvard Medical School Medical Language Program. *BMC Med Educ*. 2020;20:407. https://doi.org/10.1186/s12909-020-02323-x
22. Yedidia MJ, Gillespie CC, Kachur E, et al. Effect of communications training on medical student performance. *JAMA*. 2003;290(9):1157-1165. https://doi.org/10.1001/jama.290.9.1157
23. Romero Arocha SR, Theis-Mahon N, Ortega P. Two decades of medical Spanish education: a narrative review. *Teach Learn Med.* Published online August 13, 2024. https://doi.org/10.1080/10401334.2024.2390017
24. Ortega P, Moxon NR, Chokshi AK, Pérez-Cordón C, Park YS. Validity evidence supporting the Comunicación y Habilidades Interpersonales (CAI) scale for medical Spanish communication and interpersonal skills assessment. *Acad Med*. 2021; 96(11)(suppl):93-102. https://doi.org/10.1097/ACM.0000000000004266
25. Diamond LC, Gregorich SE, Karliner L, et al. Development of a tool to assess medical oral language proficiency. *Acad Med*. 2023;98(4):480-490. https://doi.org/10.1097/ACM.0000000000004942
26. Ortega P, Diamond L, Aleman MA, et al. Medical Spanish standardization in U.S. medical schools: consensus statement from a multidisciplinary expert panel. *Acad Med*. 2020;95(1): 22-31. https://doi.org/10.1097/ACM.0000000000002917
27. Ortega P. *Spanish and the Medical Interview: A Textbook for Clinically Relevant Medical Spanish*. 2nd ed. Elsevier; 2015.

Received: October 13, 2024
Accepted: February 28, 2025
Published: April 17, 2025

Copyright © 2025 Mai et al. This is an open-access publication distributed under the terms of the Creative Commons Attribution license.
